# MOFs based on the application and challenges of perovskite solar cells

**DOI:** 10.1016/j.isci.2021.103069

**Published:** 2021-08-30

**Authors:** Minghai Shen, Yunyu Zhang, Hui Xu, Hailing Ma

**Affiliations:** 1 School of Chemical and Environmental Engineering, China University of Mining & Technology (Beijing), Beijing 100083, China; 2 Xiamen University, Xiamen 361005, China; 3 Department of Materials Science and Engineering, University of Sheffield, Sir Robert Hadfield Building, Mappin Street, SheffieldS1 3JD, UK

**Keywords:** Energy Resources, Energy storage, Energy materials, Devices

## Abstract

In recent years, perovskite solar cells (PSCs) have attracted much attention because of their high energy conversion efficiency, low cost, and simple preparation process. Up to now, the photoelectric conversion efficiency of solar cells has been increased from 3.8% to 25.5%. Metal–organic skeleton-derived metal oxides and their composites (MOFs) are widely considered for application in PSCs due to their low and flat charge/discharge potential plateau, high capacity, and stable cycling performance. By combining MOFs and PSCs, based on the composition materials of perovskite film, electron transport layer, hole transport layer, and interfacial interlayer of PSCs, this article discusses the photovoltaic performance or structure optimization effect of MOFs in each function layer, which is of great significance to improve the photovoltaic performance of the cell. The problems faced by MOFs on perovskite solar cells are summarized, the next research directions are discussed, and the development of this crossover area of MOFs–PSC is foreseen to accelerate the comprehensive research and popularization of MOFs on PSCs.

## Introduction

So far, perovskite devices with higher efficiency are all based on organic–inorganic hybrid perovskites, and the relatively hot perovskite materials with better properties all contain organic ions. External factors, such as humidity, temperature, pressure, light, electric field, and chemical environment, strongly influence the characteristics and functions of the perovskite absorber layer. The degradation of perovskite device performance is directly related to its structural and material instability. In general, the main factors affecting device stability can be summarized as intrinsic stability of perovskite material, ion migration stability in device, and device component stability. The intrinsic stability of perovskite materials mainly includes wet stability, thermal stability, and phase stability. When the organic–inorganic hybrid perovskite is exposed to a humid environment, water molecules first diffuse into the perovskite membrane to form hydrogen bonds with volatile organic components, forming a reversible monohydrate phase. The increase of water content subsequently leads to the irreversible permanent loss of the dihydrate phase and organic molecules, and eventually the material degrades into PbX_2_ and volatile substances (CH_3_NH_2_, HI, NH_3_, I_2_, and CH_3_I) (Zhu et al., 2016; Bag et al., 2015; Ahn et al., 2016). When coupled with light/heat, the formation of PbX_2_ phase is accelerated, and the degradation rate is faster due to moisture entry (Milot et al., 2015). Application of an electric field in a humid environment will also accelerate the degradation because the loosely bound cation drift in the hydrated phase causes the perovskite structure to destabilize (Domanski et al., 2017). In addition to moisture, the superoxide (O^2−^) produced by the interaction between light-excited electrons and O_2_ molecules in the air will also react with the organic part of the perovskite to cause severe degradation (Nie et al. 2016; Aristidou et al., 2015; Pearson et al., 2016). Fortunately, the degradation caused by the combination of light and oxygen can be isolated by a hydrophobic transport layer. In addition to the influence of humidity, thermal stability is also fatal to the actual application of devices, especially perovskite materials containing organic components (such as MAPbI_3_). It was reported that MAPBI_3_ stability decreased after 24 hr of operation under 85°C and full daylight (Conings et al., 2015). In contrast, the thermal decomposition temperature of the perovskite containing FA organic molecules is 50°C higher than that containing MA (Hanusch et al., 2014).

To further improve the chemical stability and thermal stability, PSCs doped with MOFs have been developed (Makhanya et al., 2021; Chueh et al., 2019; Lee et al., 2019). Metal–organic frameworks (MOFs), also known as porous coordination polymers (PCPs), usually refer to the crystalline materials with periodic infinite network structure formed by metal ions or metal clusters and organic ligands through the self-assembly process. Therefore, it has the characteristics of both organic polymers and inorganic compounds (Liu et al., 2021). In recent decades, as a new research field, MOF compounds have shown their unique physical and chemical properties and great potential application value in many aspects such as magnetism, fluorescence, nonlinear optics, adsorption, separation, catalysis, hydrogen storage, and so on (Kandiah et al., 2010; Vieth and Janiak, 2010; Panella et al., 2010; Liu et al., 2021). Researchers can prepare functionalized MOFs for specific purposes by changing the types of ligands, modifying the functional groups in the ligands, and doping with different metal ions. Therefore, MOF materials have been favored by researchers in many fields (Stock and Biswas, 2012; Guo et al., 2020b; Gao et al., 2019). The application of MOF materials in PSC has also been widely discussed (Chueh et al., 2019; Heo et al., 2020; Yadav et al., 2020; Kaur et al., 2016).

**Figure 1:**
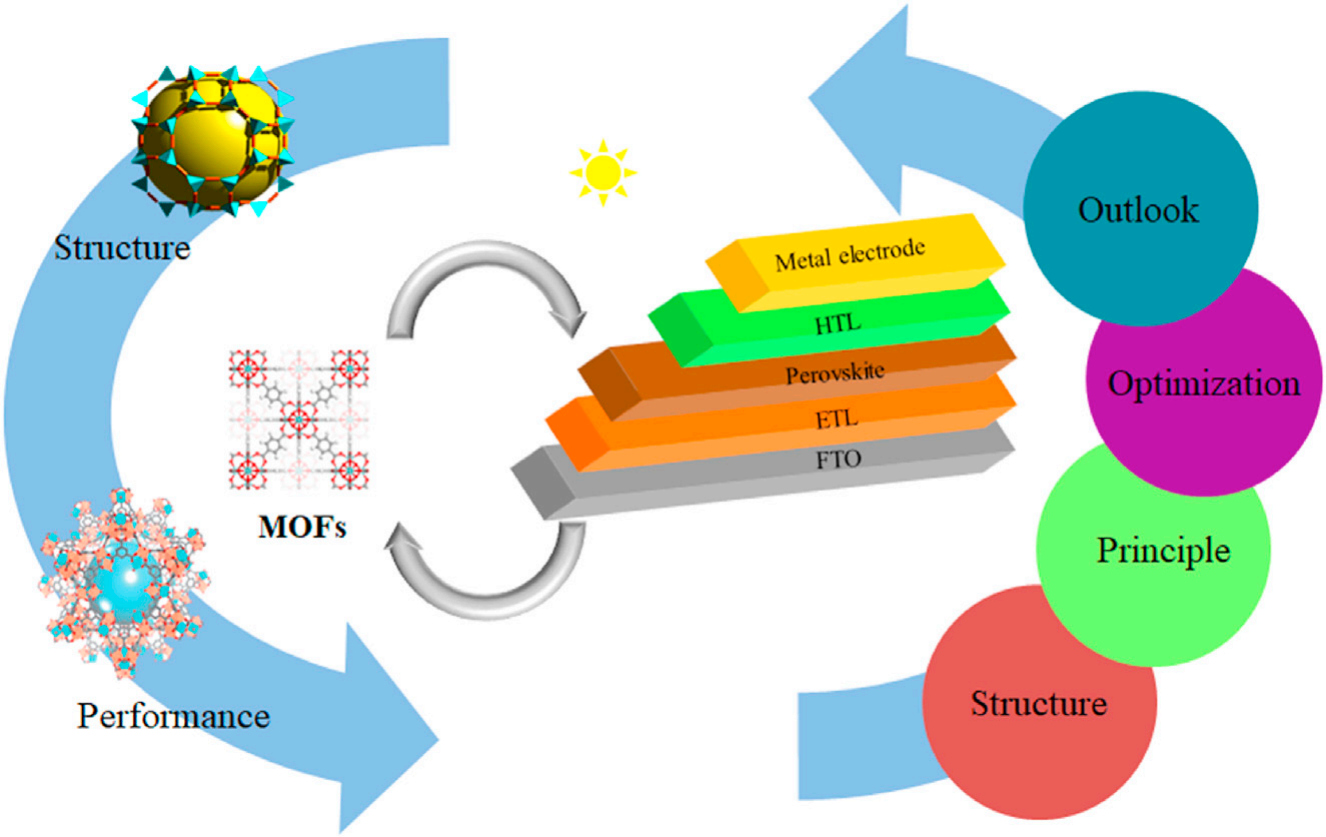
Schematic diagram.

This article summarizes the MOF materials used in PSC, and first briefly summarizes the composition and basic working principles of PSC. Then, by introducing the development of MOF materials, based on the composition materials of the perovskite film, electron transport layer (ETL), as shown in Figure 1, hole transport layer (HTL), and interfacial interlayer of the PSC, the optimization effect of MOFs on the photovoltaic performance or structure of each functional layer is discussed. Finally, it summarizes the problems that MOFs face in PSCs, discusses future research directions, and foresees the development of a cross-cutting field of MOFs-PSCs to accelerate the comprehensive research and application of MOFs in PSCs.

## Basic structure and working principle of PSC

### Composition of PSC

**Figure 2:**
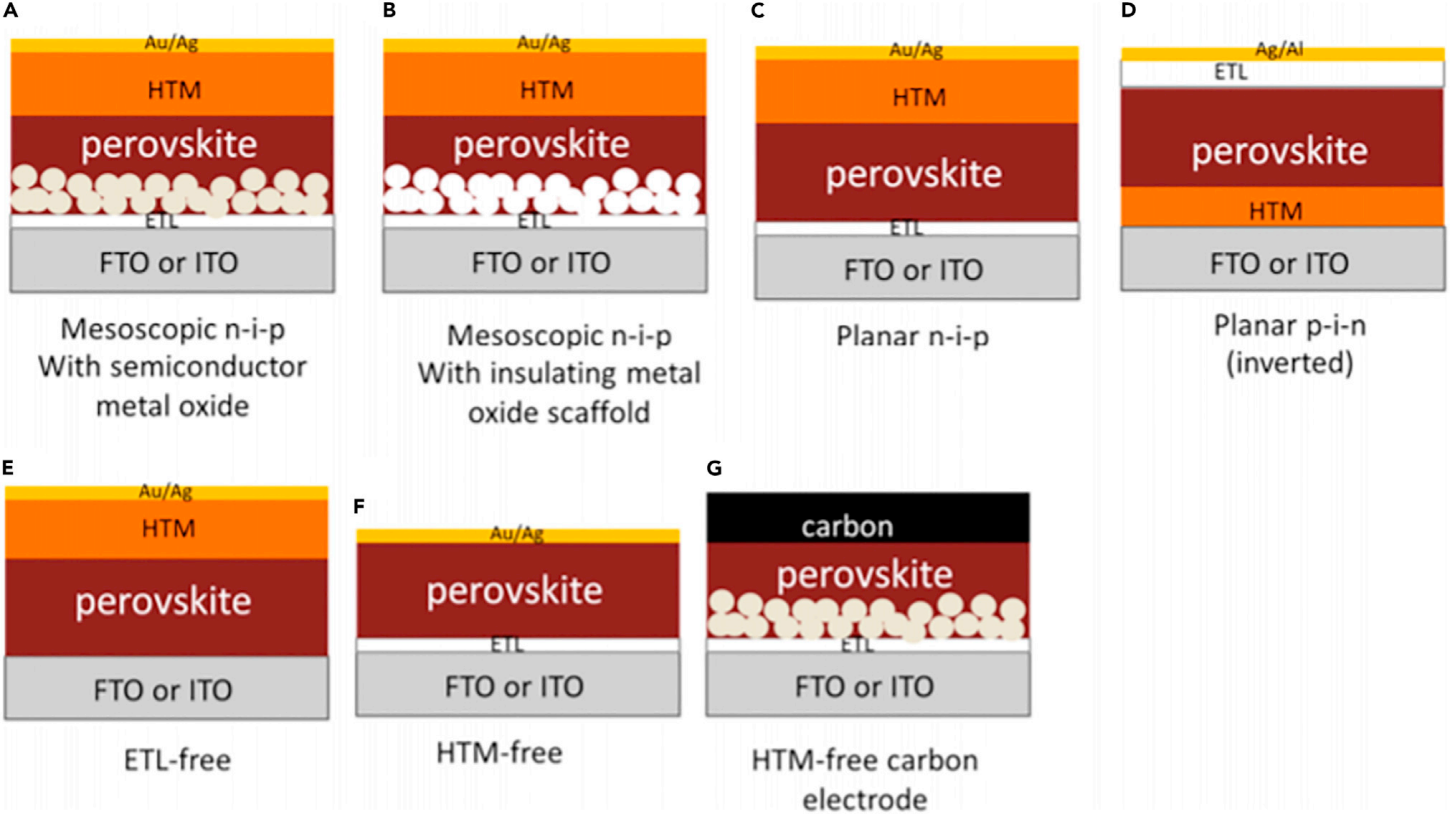
Diagrams of PSC with various structures (Jena et al., 2019).

Generally speaking, perovskite cells have the following four structures: the structure based on mesoporous n-type semiconductors, the structure based on mesoporous insulating metal oxides, the frontal planar heterojunction structure, and the transplanar heterojunction structure. These typical structures of the cell are shown in Figure 2 below. In mesoporous structures, perovskite is infiltrated into a mesoporous skeleton, which can be an n-type semiconductor or an insulating metal oxide. Above the mesoporous framework are the perovskite HTL and electrode, and below the mesoporous framework is a dense hole barrier layer and the conductive substrate. The mesoporous framework helps to form a uniform perovskite film and can also play a role in transporting electrons. In the frontal planar structure, the perovskite is surrounded by a non-porous electron and HTL to form a solar cell with an n-i-p structure. In the transplanar structure, the perovskite layer is deposited on the hole transport material, such as PEDOT, PSS, PTAA, etc., and then the ETL and electrode are deposited on the perovskite material.

Gold, silver, and carbon materials are common electrode materials in perovskite cells, but studies have shown that silver is easily corroded by reacting with the perovskite absorption layer. Common conductive substrates include ITO (indium-doped tin oxide), FTO (fluorine-doped tin oxide), etc. Among them, polymer substrates ITO-PET and ITO-PEN can be used to prepare flexible perovskite cells.

Usually in a PSC with an n-i-p structure, TiO_2_ (or SnO_2_) is directly deposited on the FTO conductive glass substrate as a commonly used ETL, and the perovskite light absorption layer (or mesoporous TiO_2_ layer plus calcium Titanium layer) is deposited on the ETL, while the HTL (usually Spiro-OMeTAD, HTM layer) is deposited on the light absorption layer, and finally a layer of the gold electrode is evaporated as the upper electrode of the PSC (Roldán-Carmona et al. 2015; Baena et al. 2015; Marchioro et al., 2014; Jiang et al., 2016). For a PSC with pin structure, the conductive substrate is generally ITO conductive glass, and the HTL is directly deposited on ITO. The hole transport material is generally PEDOT: PSS or NiOx, etc., the perovskite light absorption layer and PCBM ETL are deposited, and finally, Ag electrodes are prepared (Jin et al., 2015; Chiang and Wu, 2016; Chen et al., 2018a). In the PSC structure described above, the incident light is incident from the glass surface, passes through the glass and conductive oxide electrode (FTO/ITO), passes through the electron or HTL, is absorbed in the perovskite layer, and finally reaches the upper electrode of the cell. No matter what kind of device structure the PSC belongs to, its most important part is the perovskite light absorption layer.

### Power generation principle of PSC

The photoelectric energy conversion process in a solar cell has two necessary steps: First, it absorbs light energy and produces electron–hole pairs; Second, the device structure disconnects electrons and holes and conducts them away. The electrons flow to the negative electrode and the holes flow to the positive electrode, forming voltages and currents. Therefore, the generation, dissociation, transport, and recombination of electron–hole pairs can be considered to improve the photoelectric conversion efficiency of solar cells.

(1) Light absorption and the generation of electron–hole pairs.

**Figure 3:**
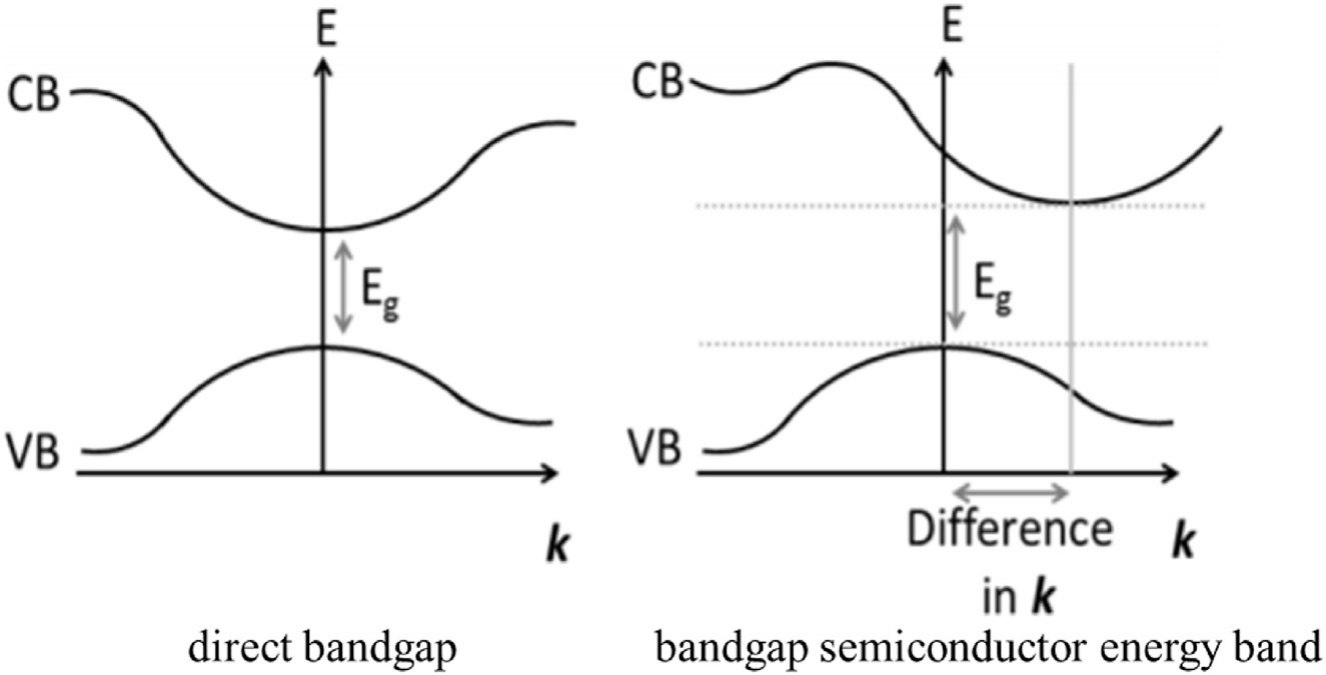
Schematic diagram of the direct bandgap and the bandgap semiconductor energy band structure (Eperon, 2015).

When light is irradiated to the light-absorbing material of the solar cell, the intrinsic absorption between the energy bands will cause the electrons of the light absorbing material to transition from the valence band to the conduction band, leaving positively charged holes in the valence band, thus producing an electron–hole pair. This process requires that the energy of the incident photon is not less than the forbidden bandwidth E_g_ of the light-absorbing material, that is, h_ν_≥ E_g_= h_ν_ or h_c_/λ_0_≥ E_g_= h_c_/λ_0_, to make the electron transition from the valence band to the conduction band, ν_0_ and λ_0_ are the frequency and wavelength of the critical intrinsic absorption photon. In theory, the larger the intrinsic absorption wavelength range of the semiconductor material, the more electron–hole pairs that can be generated, and the higher the PCE of the corresponding solar cell. The intrinsic light absorption process of light-absorbing materials needs to satisfy both the conservation of energy and the conservation of momentum. Specifically, as shown in Figure 3, the conduction band bottom of a direct band-gap semiconductor has the same wave vector k as the valence band top. When the electrons in the valence band transition to the conduction band, their momentum remains unchanged; it is a direct transition. By analyzing the perovskite crystal structure of the indirect band, the bottom of the conduction band and the top of the valence band of the semiconductor correspond to different wave vectors k. When the electrons in the valence band transition to the conduction band, to satisfy the momentum conservation relationship, the momentum of the electrons will change, accompanied by the absorption or emission of phonons related to the lattice vibration. This process is called the indirect transition. Therefore, in addition to electron–photon interaction, the indirect transition process is accompanied by electron-phonon interaction, which makes the light absorption coefficient of the indirect band-gap semiconductor material significantly lower than that of the direct band-gap semiconductor. Thinner direct bandgap semiconductor film can absorb more spectral energy. Organic–inorganic metal halide perovskite is a direct bandgap semiconductor material with excellent light absorption performance. The bandgap of a typical CH_3_NH_3_PbI_3_ perovskite is ∼1.55 eV, and its self-generated exciton binding energy is only 19 ± 3 meV (Sun et al., 2013), the valence band electrons are easily excited to the conduction band, leaving vacancies in the valence band to form electron–hole pairs.

(2) Dissociation and transport of carriers.

**Figure 4:**
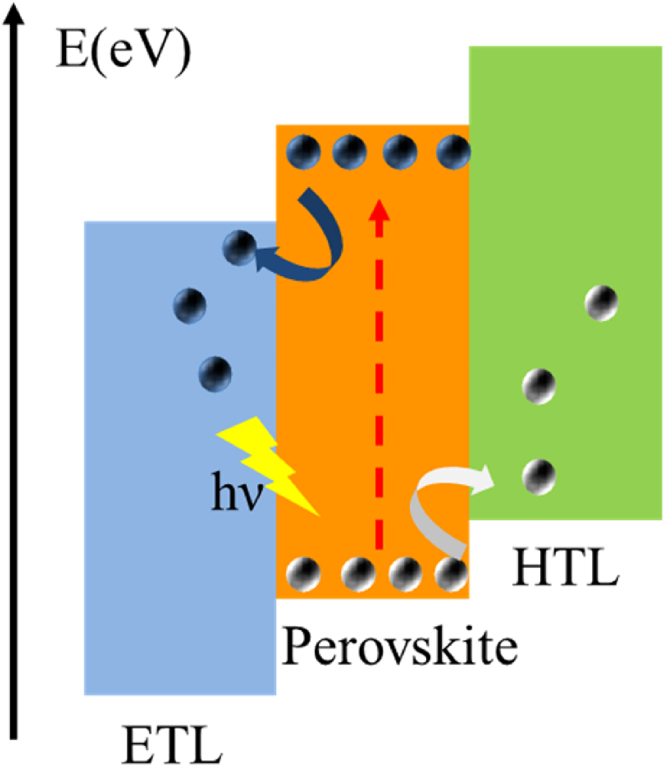
Schematic diagram of working principle of PSC.

Due to the energy gap between the perovskite and the ETL and the HTL, the photogenerated electron–hole pairs will be dissociated and injected into the ETL and HTL respectively, and then a photocurrent and a photovoltage after being collected by the respective electrodes. The process is shown in Figure 4. The carrier mobility of the perovskite material itself is relatively high, and the diffusion distance is long. For example, the carrier diffusion length of CH_3_NH_3_PbI_3_ is about 100 nm, and the carrier diffusion length of CH_3_NH_3_PbI_3_-xClx can exceed 1 μm (Stranks et al., 2013), which is very beneficial to the dissociation and transport of carriers. Of course, there will inevitably be losses in the process of carrier transport, such as the recombination of holes in the perovskite layer and electrons in ETL, the recombination of electrons in the perovskite layer and holes in HTL, and the recombination of electrons in ETL and holes in HTL (The perovskite layer has defects such as holes), as well as the trapping and annihilation of carriers by defects on the surface and interface, etc., which will have a negative impact on the photoelectric performance of the device. How to improve the yield of electron–hole pairs and reduce the recombination loss requires consideration from the aspects of improving the crystal quality of the perovskite layer, optimizing the surface and interface of the device, and reducing defects.

## MOFs based on the application of PSC

**Figure 5:**
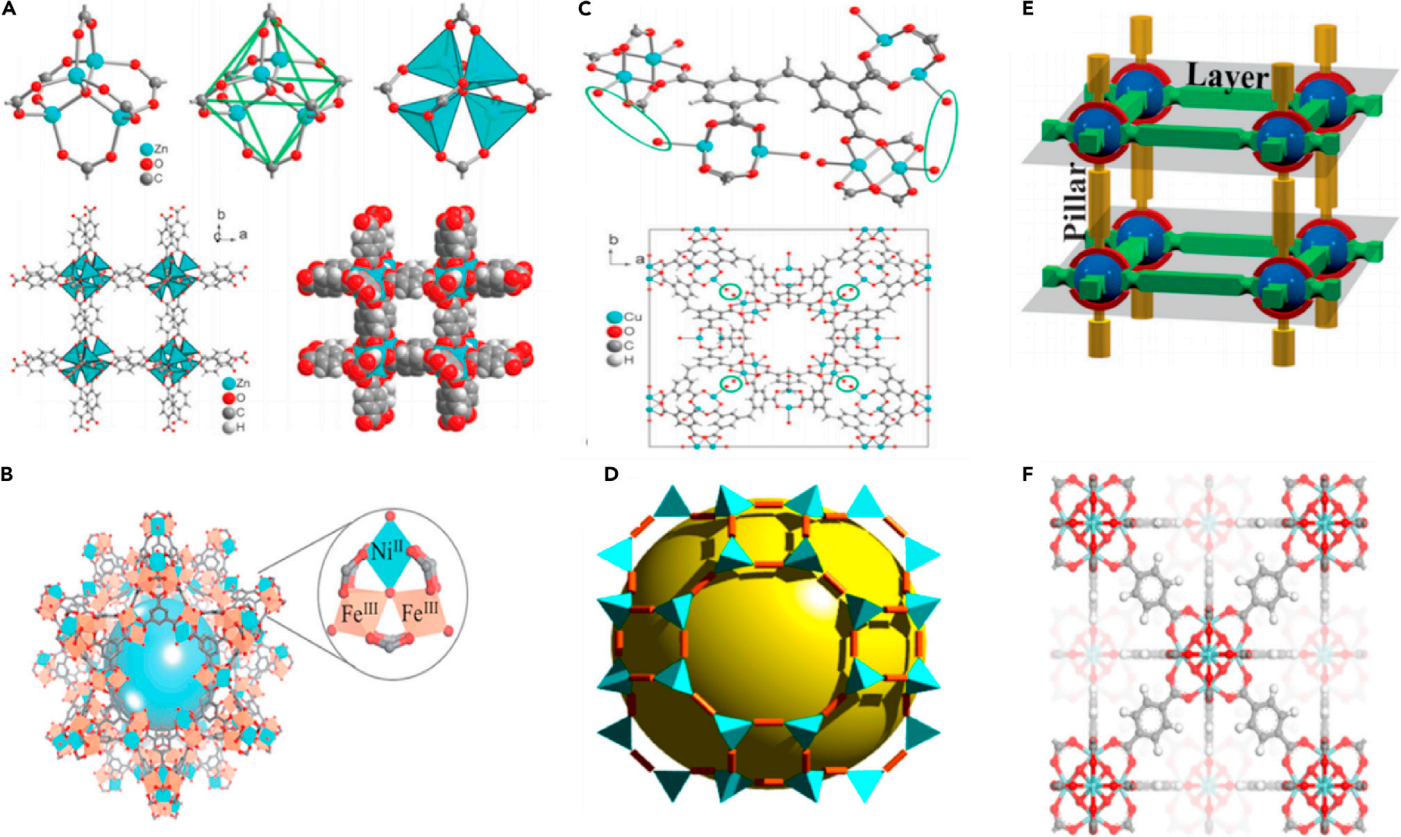
3D molecular structures of various MOFs (A) MOF-5 (IRMOF-1), 3D-[Zn_4_O(bdc)_3_] (Vieth and Janiak, 2010). (B) MIL-100(Fe, Ni) (Giménez-Marqués et al., 2019). (C) PCN-12(Cu) (Vieth and Janiak, 2010). (D)ZIF-71(Zn) (Fei et al., 2013). (E)CPLs-55(Zn) (Hu et al., 2015). (F) UiO-66(Zr) (Kandiah et al., 2010).

There are many types of MOF materials. According to their different crystal structures and different research groups, they can be divided into the following categories: IRMOFs (Rosi, 2003), MILs (Ferey et al., 2005), PCNs (Ma et al., 2008), ZIFs (Wang et al., 2008; Phan et al., 2010), CPLs(Coordination Pillared-Layers) (Seo et al., 2009),UIOs (University of Oslo) (Cavka et al., 2008; Yang et al., 2011), etc. The three-dimensional structure is shown in Figure 5.

(1) IRMOF series

IRMOFs are a series of MOFs with the same or similar crystal structure discovered and named by the Yaghi research (Eddaoudi, 2002). IRMOF series crystal materials are organo-metallic skeleton materials formed through self-assembly with W octahedral [ZnO_4_]^6+^ ion clusters as nodes and symmetrical organic dicarboxylic acid ligands as bridges. The pore size of this series of materials is mainly determined by the length of the organic ligand connected to the metal node. To adjust the pore size of the MOF material to be suitable for different reaction occasions, scientists led by Yaghi have successively discovered a series of IRMOF-N materials with different pore sizes and studied their pore size characteristics and adsorption properties.

(2) MIL series

MILs are a series of MOF materials discovered and named by Férey’s research (Millange et al., 2002). This type of material is mainly divided into two categories: one type is MOF materials synthesized by self-assembly; among this type, transition metals as nodes, succinic acid, glutaric acid, and other dicarboxylic acid organic ligands as bridges. One of their unique properties is the “respiration phenomenon”, which is manifested in the flexibility when adsorbing polar molecules such as water or carbon dioxide, and the reversible transformation of small pores and large pores in the material structure (Surble et al., 2006; Serre et al., 2004; Loiseau et al., 2010). The other type is valence transition metal ions (such as Fe^3+^, Al^3+^, Cr^3+^, and V^3+^, etc.) as the nodes, and carboxylic acid organic ligands such as terephthalic acid or succinic acid as the bridges and synthesized a series of MOF materials with MTN topological structure through coordination between groups. Analyzing the structural characteristics of this type of material, it is found that they all have a rigid cage structure, and have two mesoporous-scale pore structures with different diameters, which are called large and small pore structures (Férey et al., 2010).

(3) PCN series

PCNs are a series of MOF materials composed and named by the Zhou (Wang et al., 2007; Sun et al., 2006). PCNs are mainly composed of bivalent copper ions as nodes and H3TATAb and HTB(S-Heptazine teribenzoate) as bridges, which are metal–organic skeleton materials formed through self-assembly. The crystal structure of this material contains multiple three-dimensional pore structures composed of regular octahedrons and has strong adsorption capacity. Among them, PCN-14 has a considerable amount of methane adsorption and storage and has certain application potential in energy storage (Ma et al., 2008).

(4) ZIF series

ZIFs are a series of MOFs materials discovered and named by the Yaghi research (Park et al., 2006). ZIFs are mainly MOF materials synthesized by self-assembly with transition metal ions such as zinc or cobalt as nodes and imidazoles and their derivatives as bridges. The material has a structure similar to zeolite molecular sieve, high thermal stability, strong adsorption capacity, and broadens the types of organic ligands in the synthesis of MOFs (Hayashi et al., 2007).

(5) Other MOFs

CPLs are a series of MOF materials discovered and named by the Kitagawa research (Kondo et al., 1999). CPLS is a MOF material synthesized by self-assembly between groups using divalent transition metal ion clusters as nodes and nitrogen-containing heterocyclic organic ligands such as 2,2′-bipyridine, 4,4'-bipyridine or phenol as bridges. UiOs are a series of MOF materials discovered and named by the Lillerud research group (Cavka et al., 2008). Among them, the most valuable material is UiO-66 (Zr). The crystal material, the metal cone ion cluster as the node and terephthalic acid as the bridge, synthesized MOFs through the self-assembly between groups. The material contains two-pore structures with different diameters, which has potential application prospects in gas adsorption, separation, and capture (Zhang et al., 2012).

In addition to the synthesis of new MOFs, the modification of MOFs is also a major research trend at present. Many studies are based on the original MOFs, through appropriate adjustment of reaction conditions to change a certain characteristic of MOFs, to achieve a certain purpose (Meilikhov et al., 2010; Xiang et al., 2011; Lan et al., 2010).

### Perovskite film

Since the perovskite layer is the main place where the perovskite solar cell undergoes photoelectric conversion and carrier dissociation and transport, the morphological quality of the perovskite film is very important to the performance of the cell device (Xiao et al., 2019). During the crystallization process of organic–inorganic halide perovskite, the crystal nucleus does not grow uniformly, so researchers have improved the morphology quality of the film by adjusting crystallization (including nucleation and growth) or post-passivation and other post-processing methods (Shahbazi and Wang, 2016; Huang et al., 2018). Although the organic–inorganic hybrid MAPbI_3_ perovskite has been extensively developed in recent years and its efficiency has been greatly improved, the instability of its components has always been a problem that plagues scholars. In the working process, MAPbI_3_ is easy to decompose under the action of water, oxygen, heat, and light. Using inorganic Cs^+^ ions instead of MA^+^ ions to fill the A-site voids can improve the instability of its composition. However, the too small ion radius of Cs^+^ ions makes it difficult to support the perovskite framework of PbI_6_, causing the lattice to be twisted and distorted, resulting in the formation of δ-phase without photovoltaic characteristics (Zhou et al., 2019; Abdelmageed et al., 2016).

**Figure 6:**
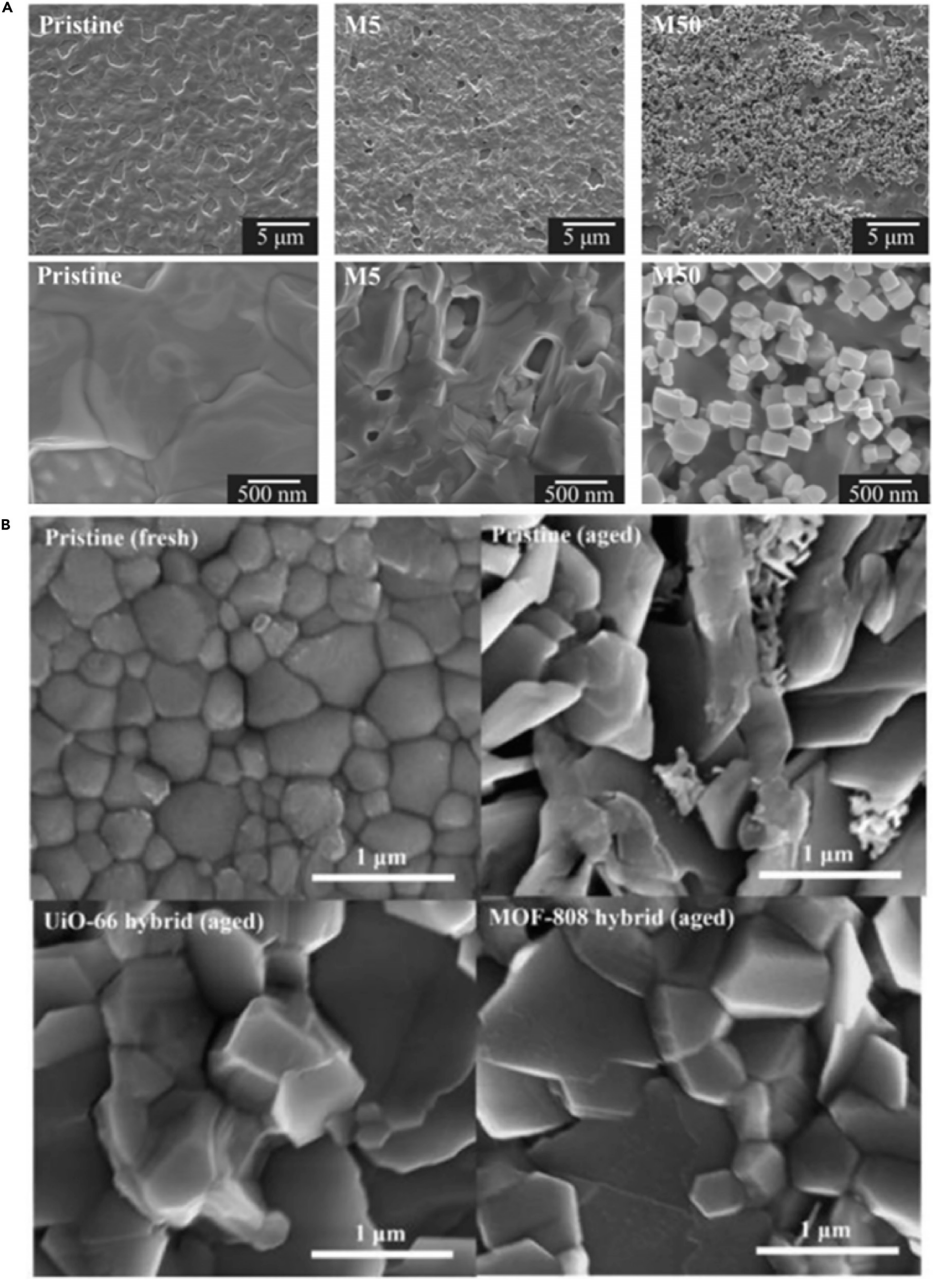
SEM of perovskite films after different aging times (A) modified M5 and M50 films (Chang et al., 2015). (B) UiO-66 hybrid and MOF-88 hybrid films (Leeet al., 2019).

**Figure 7:**
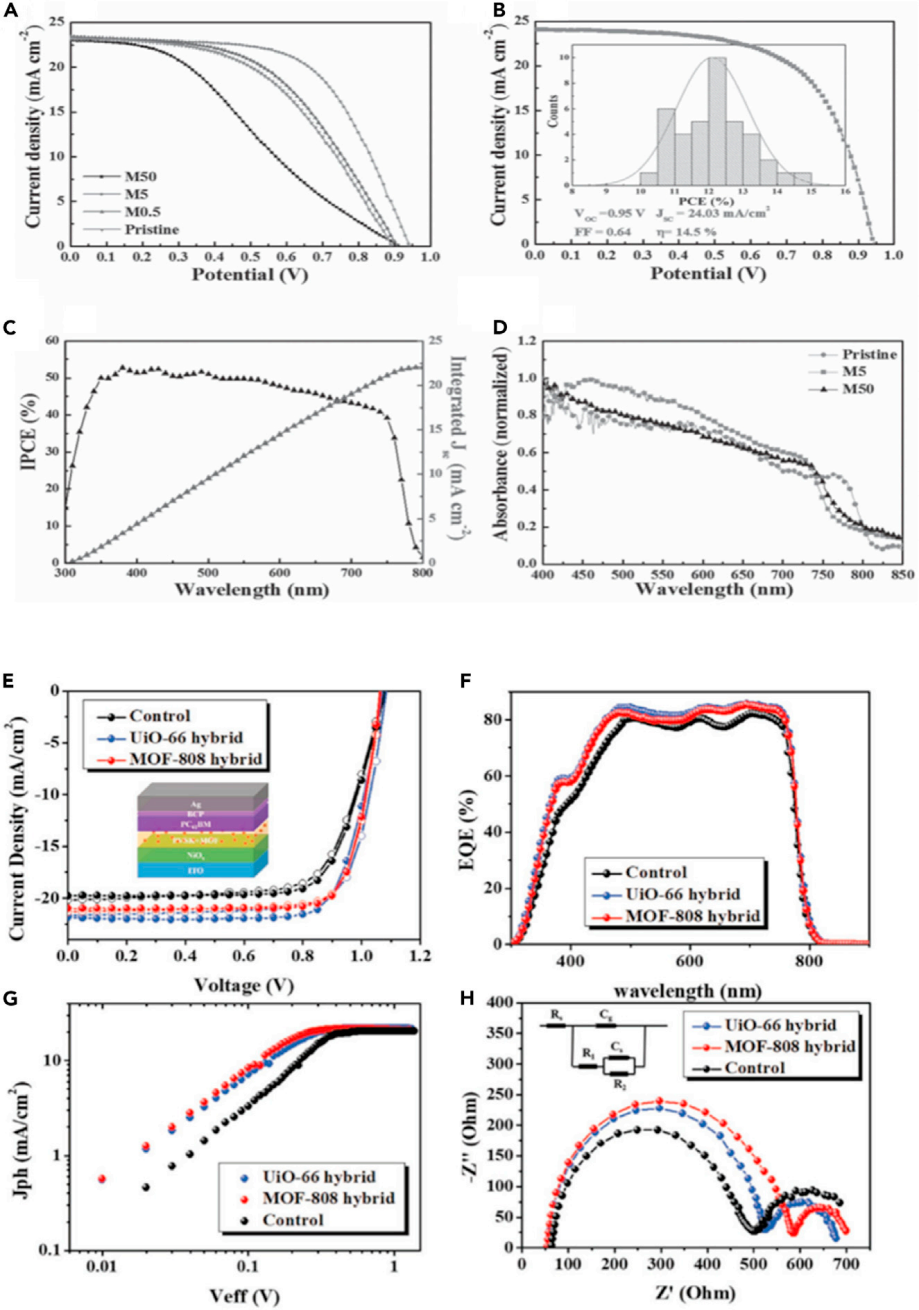
Correlation spectrum of MOFs/PSC with current density plot (A) J–V curves of the perovskite and MOF/PSC. (B) J–V curve of the best M5 cell. The inset is the histogram of average efficiencies for 40 devices. (C) IPCE spectrum and the integrated photocurrent density of the M5 cell. (D) UV–visible absorption spectra of the perovskite and MOF/perovskite thin films (Chang et al., 2015). (E) The J–V curves. (F) The EQE spectra. (G) Jph–Veff characteristics. (H) The EIS analysis of the studied MOF-hybrid PVSCs (Leeet al., 2019).

With the use of mesoporous structures, solid perovskite with a PCE exceeding 10% can be developed. Polymetallic sulfides with the same topological structure as inorganic perovskites have interesting properties, such as the coexistence of ferroelectric and magnetic order. These concepts have been conceptualized in the recent development of PSCs. As one of the early examples in this regard, Chu has combined the small crystal size of MOF-525 with perovskite to significantly enhance the morphology and crystallinity of the perovskite film (Chang et al., 2015). By adding MOF-525 microporous nanocrystals as an additive, the morphology and crystallinity of the perovskite film have been significantly improved, as shown in Figure 6A. Micro-porous MOF-525 nanocrystals are mixed near the bottom of MOF/perovskite composite membrane, and used as a conventional scaffold, so that perovskite crystals occur inside. Therefore, this traditional scaffold provides an orderly arrangement of perovskite microcrystals during the initial stage of crystallization. As shown in Figure 7 (ad), it can be observed that the M 5 cell performs best among all solar cells, with an average PCE of 12.0%, an open-circuit voltage (Voc) of 0.93 V, and a short circuit current density (*J*_sc_) of 23.04 mA cm^−2^, The fill factor (FF) is 0.60; the cell performance is significantly better than the control cell made from the original perovskite precursor solution (average PCE = 10.1%). It should be noted that compared to the original cell, the M5 cell shows significantly improved Voc and FF, while the *J*_sc_ is more comparable. However, the *J*_sc_ and FF of the M50 cell are significantly lower. Under AM 1.5G illumination, the M5 solar cell with the best performance can obtain a Voc of 0.95 V, a *J*_sc_ of 24.03 mA cm^−2^, an FF of 0.64, and a PCE of 14.5%.

Fan purpose-synthesized microporous In (in-based MOF) BTC nanocrystals, and the perovskite/in-BTC heterostructure demonstrated its effectiveness as a light-collecting layer for achieving efficient and long-term stable PSC (Zhou et al., 2020). In-BTC additives can improve the morphology and crystallinity of the perovskite while reducing the grain boundaries and defects of the perovskite film (Lee et al., 2016), as shown in Figure 6B. Through optimized interface electrical contact and light response, the perovskite/In-BTC heterojunction PSC has obtained enhanced PCE (19.63 ± 1.24%). Also, after placing IN-BTC-modified PSCs in a high humidity environment for 12 days without packaging, it can retain more than 80% of the initial PCE, which exceeds the remaining 35.4% of the original cell. This shows that the use of perovskite/In-BTC heterojunction as the perovskite film to produce an effective and stable PSC is a simple and effective strategy. The PV parameters of the perovskite thin film of MOFs applied to PSC are shown in Table 3.

Although MOFs are currently less studied in the perovskite thin films of PSC, their excellent performance in solar cells proves their application prospects. Wu used perovskite/Zr- MOF heterojunctions, which proved the potential of using perovskite/MOF heterojunctions to produce efficient and stable PVSC (Lee et al., 2019). Zr-MOF, namely UIO-66 and MOF-808, shows the advantages of UV-filtering capability and enhanced perovskite crystallinity in the MOF used as an interlayer with perovskite membranes in achieving efficient, stable, inverted P-I-N.

PSC (PVSC). The power conversion efficiency (PCE) of PVSC modified by UIO-66/MOF-808 increased by 17.01% and 16.55%, respectively, which was better than that of the control device (15.79%).In these devices, it was found that the mixed MOF may be distributed along the perovskite grain boundary, providing a solid crystal effect that simultaneously passivates the defect and enhances the moisture resistance of the film. The results showed that the PCE of UIO-66/MOF-808 mixed PVSC was further increased to 18.01% and 17.81%, respectively, as shown in Figures 7E–7H.

The electron–hole binding energy of the perovskite material itself is very low, which makes it easy to separate electron–hole pairs under light conditions, improving the efficiency of the PSC from another angle. Compared with many semiconductor materials, the perovskite material film does not have many internal defects, which provides conditions for it to work efficiently, and the high mobility makes the perovskite material easier to generate photocurrent. It is these excellent material properties that keep perovskite-based solar cells hot. At present, the efficiency of PSCs has made rapid progress. However, high-efficiency perovskite cells are mainly derived from the small area of the laboratory and are far below the theoretical prediction value. Therefore, the preparation of large-area high-efficiency devices is a major challenge for commercialization. The preparation of high-quality, low-defect, large-area perovskite films is the primary prerequisite for realizing high-efficiency, large-area PSCs, and it is also an urgent problem to be solved at present. High-efficiency PSCs are based on polycrystalline thin-film cells, and there are a large number of defects in the grain boundaries (GBs), interior, and interfaces of these polycrystalline thin films. For example, the volatilization of the solvent during the film annealing will leave a certain amount of defect at the interface, and the non-stoichiometric ratio will also produce defects during the manufacturing process. These defects act as charge recombination centers, leading to serious carrier recombination during device operation, destroying cell efficiency and stability (Shao et al., 2014; Ye et al., 2016; Jiang et al., 2019; Wanget al., 2016; Li et al., 2016). Theoretical studies further show that the defects of perovskite films mainly come from uncoordinated halide ions and halide-rich anti-site defects (de Quilettes et al., 2015; Leblebici et al., 2016; Kutes al., 2016; Bischak et al., 2015). However, the defect density in the grain boundary of the highly efficient perovskite devices that prepare polycrystalline perovskite films based on the low-temperature solution is 1016 cm^−3^, which is several orders of magnitude higher than that of single-crystal films (Huang et al., 2015; Wei et al., 2016; Fang et al., 2015). Therefore, effective passivation of perovskite thin film is one of the necessary guarantees to achieve high efficiency and stability of PSC. Studies (He et al., 2019; Lee et al., 2019; Ji et al., 2021) have shown that MOF can improve the effective passivation of the perovskite surface. For perovskite films, mixed MOF may be distributed on the perovskite grain boundaries to provide a grain locking effect, passivate defects and enhance the film’s robustness to moisture intrusion (Lee et al., 2019).

### Hole transport layer

Although the perovskite material itself has electron–hole dual transport properties to achieve higher photoelectric conversion efficiency, the role of the HTL cannot be ignored. It can be seen from the working principle of the above PSCs that the HTL is very important in photovoltaic devices. HTL is not only responsible for transporting holes and preventing direct contact between the perovskite layer and the conductive glass surface, but it also has an important influence on the crystal orientation, grain size, and morphology defect of perovskite crystal (Calió et al., 2016). In these processes, the HTL materials mainly play the following four roles: (1) Promoting the separation of electrons and holes; (2) Forming an ohmic contact with the perovskite layer to effectively collect holes; (3) Effectively transporting holes to the electrode; (4) Effectively blocking electrons from entering the HTL. Therefore, excellent hole transport materials can effectively avoid the loss of holes during the transport process and have an important impact on the performance of PSCs. With the rapid development of PSCs, the study of HTL materials is an important research direction in the field of PSC (Murali et al., 2015). To find the most suitable hole transport material in the p-type material, the molecular HOMO energy level of the material should be slightly lower than the valence band of the perovskite. On the other hand, the free energy of the hole should match the Fermi level of the hole transport material and the perovskite. At the same time, holes have excellent transport capacity (>10^−3^ cm^2^V^−1^s^−1^) and good thermal stability (Habibi et al., 2016).

Commonly used hole transport materials can be divided into two types: organic and inorganic. Organic materials also include polymer materials and small molecule materials. The cost of small molecules is relatively lower, the processing method is more flexible, and the bandgap is adjustable to make it easier to match the material. In addition, the polymer material has higher hole mobility, it is not easy to crystallize, and has a better surface coverage.

At present, the commonly used organic hole transport materials mainly include Spiro-OMeTAD (Min et al., 2019), PEDOT:PSS (Jeng et al., 2013), PTAA (Deng et al., 2018), and so on. However, organic materials are generally more expensive and have relatively poor stability. Organometallic compounds can also be used as HTLs, such as copper phthalocyanine (Cu Pc). Georgia et al. used tetramethyl substitution to improve the performance of copper phthalocyanine. They speculated that methyl substitution could enhance the interaction of the Π–Π bond, but the efficiency was only 5% (Sfyri et al., 2016). Yan et al. used C60 as the ETL and evaporated 60 nm copper phthalocyanine to prepare a cell with an efficiency of 15.42%. This method can also be used in flexible solar devices and achieved a conversion efficiency of greater than 7% (Ke et al., 2015). After 1000 hr of the aging test, it is found that this cell is more stable than the cell based on Spiro-OMeTAD. In the unpackaged case, the efficiency of the cell based on Spiro-OMeTAD decreased by 38.65% from the initial value, while the cell based on copper phthalocyanine only decreased by 2.78%. Suzuki was the first to use lead phthalocyanine, zinc phthalocyanine, and cuprous thiocyanate as the HTL to prepare PSC. The HOMO positions of these three materials were −5.0 eV,−5.2 eV, and −5.3 eV, respectively. The efficiencies are 3.97%, 3.5%, and 1.5%, respectively, while the cell made under similar conditions without the HTL has an efficiency of 3.45% (Suzuki et al., 2015). Recently, Arora reported that the efficiency of the cell based on cuprous thiocyanide has reached 20.3%. They prepared the cuprous thiocyanide nanocrystalline film by rapidly evaporating the solvent, reducing the contact time between the solvent and the perovskite (Arora et al., 2017). Spiro-OMeTAD is the most widely used small molecule hole transport material in PSC. Pure Spiro-OMeTAD has poor hole mobility and conductivity and generally requires doping to improve performance. Undoped Spiro-OMeTAD was first used in dye-sensitized solar cells, and the conversion efficiency of the cell was only 0.7%. Subsequently, the Spiro-OMe TAD was doped with t-BP (4-tert-butylpyridine) and Li-TFSI (bis (trifluoromethulfonyl) lithium imide) to increase the efficiency to 2.56%. These two dopants can inhibit the recombination of carriers at the interface and improve the conductivity of the hole material and the open-circuit voltage of the cell (Fabregat-Santiago et al., 2009). To improve the hole mobility of Spiro-OMe TAD, Burschka et al. introduced a trivalent cobalt compound FK102 in Spiro-OMeTAD, the cell efficiency increases to 7.2% (Burschka et al., 2011). Snaith et al. used dual-source co-evaporation to prepare a uniform and flat mixed perovskite CH_3_NH_3_PbI_3−_xClx, with a cell efficiency of 15.4% (Liu et al., 2013) Seok et al. used a mixed perovskite system (FAPbI_3_) 0.85 (MAPbBr_3_) 0.15, and the efficiency of the cell prepared with Spiro-OMeTAD as the hole layer was 19% (Jeon et al., 2015). Tress et al. optimized the ratio of PbI_2_ to FAI in the mixed system perovskite and used excessive lead iodide to improve the electrical properties of the perovskite. The conversion efficiency of the prepared mesoporous structure device was 20.8%, and the open-circuit voltage of the cell reached 1.16 V (Bi et al. 2016). Sargent et al. used CL-passivation to reduce recombination at the interface (Tan et al., 2017). The cell efficiency that uses mixed perovskite Cs_0.05_ FA_0.81_ MA_0.14_ PbI_2.55_ Br_0.45_ and preparation of Spiro - OMeTAD in the flat structure is 20.1%, and the device effective area is 0.049 cm^2^. Seok et al. studied the influence of the position of the methoxy group (-OCH_3_) in Spiro-OMeTAD (Jeon et al., 2014) by analyzing the position of the methoxy group in ortho (po-OMe), meta (pm-OMe), and right (pp-OMe) The optoelectronic properties of the position. With the change of the substituted position of methoxy, the oxidation potential also changed. When the para-position changed to meso-position, the HOMO position of Spiro-Ometad changed from −5.22eV to −5.31eV, while when the methoxy group was in the ortho position, the LOMO position increased from −2.28eV to −2.18eV, which is conducive to blocking the transmission of electrons from perovskite to the gold electrode, thus improving the filling factor and efficiency. Chemical doping (Li et al., 2018; Zhang et al., 2021) through MOF may be an effective strategy to improve the performance of Spiro-OMeTAD, and most dopants are designed for controlled oxidation of Spiro-OMeTAD. The study (Wang et al., 2021) found that Zn-CBOB can not only controllably oxidize Spiro-OMeTAD and improve the conductivity of HTM, but also passivate the surface traps of the perovskite film by coordinating with Pb^2+^. The PSC with Zn-CBOB doped with HTL achieved a significant PCE of 20.64%. The hydrophobicity of Zn-CBOB can prevent water from destroying the perovskite layer, which helps to improve the stability of PSC.

Thiophenes have excellent photoelectric properties and high hole mobility, making them a fine choice for hole transport materials. Nazeeruddin et al. synthesized a thiophene derivative PST1 with a structure similar to Spiro-OMeTAD, and prepared a cell with a conversion efficiency of 13.4% by doping with cobalt salt FK209 (Ganesan et al. 2015). Zimmermann et al. synthesized a series of materials with ATT as the core and found that as the hydrocarbon-based chain grows, the solubility of the material will increase, while the conductivity and efficiency will decrease. The highest efficiency of the cell they prepared is 18.1% (Zimmermann et al., 2017). Saliba et al. synthesized another thiophene derivative FTD. The thermal and photoelectric properties of this material are very similar to Spiro-OMeTAD (Saliba et al., 2016). The efficiency of the PSC prepared by FTD reached 20.2%, the short-circuit current was 22.7 mA·cm (Bag et al., 2015), the open-circuit voltage was 1.15 V, and the fill factor was 0.76. Triphenylamines are commonly used as hole transport materials in organic light-emitting diodes (OLED), dye-sensitized cells, and organic photovoltaic cells. Ko et al. synthesized a series of triphenylamine derivatives containing diphenylvinyl sidearms and obtained a conversion efficiency of 11.8% in a cell with MAPbI3 perovskite (Choi et al., 2014). Then they synthesized the material DMFA-FA (Choi et al., 2015) with the bis-dimethyl fluorenyl amino and triphenylamine core, which has higher hole mobility and molecular stability than triphenylamine, and the prepared cell efficiency is 14.2%. Chen et al. used the triphenylamine derivative m-MTDATA (Chen et al., 2018b) to prepare a trans flat cell, its efficiency is18.12%. Tricarbazole derivatives are another class of small molecular hole materials that have been used earlier. Rakstys et al. introduced different alkoxy groups into Triazatruxenes (Rakstys et al., 2015), adjusting the HOMO position to match the perovskite. The cell efficiency of 5,10,15-trihexyl3,8,13-tris(4-methoxyphenyl)-10,15-dihydro-5Hdiindolo[3,2-a:3′,2′-c]carbazole (KR131) was 18.3%.

Except for PEDOT: PSS, most materials are easily dissolved by the perovskite solvent DMF or DMSO, so they are generally only used in cells with positive structure. The advantages of reverse plane structure PSC based on PEDOT: PSS cave-transport materials are that they can be prepared at full low temperature, and avoid the high-temperature sintering process, similar to the forward structure cell to prepare TiO_2_ thin film needed. Also, it consumes less energy and is more conducive to the preparation of flexible devices. There is still a lot of room for improvement in the cell efficiency of this structure, but there are still some difficulties in achieving high efficiency. For example, the perovskite film has pinholes in the PEDOT: PSS layer and the coverage is not complete, resulting in a decline in the optical performance of the device. Besides, the energy level matching between PEDOT: PSS and the perovskite material is not high, resulting in incomplete ohmic contact between the perovskite light absorption layer and the HTL, which in turn causes the loss of open-circuit voltage. Therefore, most of the PEDOT: PSS-based trans-planar structure cells have an open circuit voltage of less than 1 V.

The research of PSC draws on the experience of many organic photovoltaic cells. In addition to small-molecule materials such as Spiro-OMe TAD, some polymer hole materials used in organic photovoltaic cells are also used in PSC. PTAA (Poly-Triarylamine) is one of the first polymer hole materials used in perovskite cells and has achieved a very high conversion efficiency. As early as 2013, Seok et al. used PTAA as a HTL to prepare PSC with a conversion efficiency of 12% (Jeon et al., 2013). With the optimization of PTAA and the use of new hybrid system perovskite materials (Yang et al., 2015), they have increased the cell efficiency to 20%. Yang et al. used TFB to prepare a perovskite pool with an efficiency of 12.8% (Zhu et al., 2014). Yan et al. deposited three materials with simple structures by electropolymerization (Yan et al., 2015):PPP, poly(p-phenylene), PT, polythiophene, and PPN, poly(4,4′-bis(N-carbazolyl))-1,1′-biphenyl), the cell efficiency is increased to 16.5% by optimizing the film thickness. Seok et al. used copolymers PCPDTBT and PCDTBT to prepare devices, and obtained efficiencies of 5.3% and 4.2%, respectively (Heo et al., 2013). Lee et al. used PTB7-Th superimposed with MoO_3_ to prepare a flat cell with a voltage of 1.03 V and an efficiency of 11.04% (Kim et al., 2014). Yang et al. used a stack of PBDTTT-C and MoO_3_ as hole transport materials in a structure without an ETL (Chen et al., 2015), and the cell efficiency reached 9%. Also, materials such as PTB-BO (Lee et al., 2015) and PDPPDBTE(Kwon et al., 2014) have also been studied, but the conversion efficiency is not high. Most of these polymer materials only play a role in transporting charges instead of absorbing sunlight. Therefore, the development of materials with an absorption zone outside the perovskite absorption zone should improve the external quantum efficiency of the device. Poly(3-hexylthiophene) (P3HT) is also a common polymer in OPV. Zhang et al. improved the efficiency of the trans-perovskite cell prepared at the P3HT/perovskite interface by plasma treatment to 16.2% (Zhang et al., 2018). The photovoltaic parameters of various organic hole-transporting PSC are shown in Table 1.

**Table 1:** Comparison of photovoltaic parameters of PSC transported by various organic holes.

Organic material	Category	Voc(V)	*J*_sc_ (mA cm ^−2^)	FF	PCE(%)	References
CuPc	Organo-metallic compound	0.65	16.5	0.50	5.0	Sfyri et al. (2016)
CuPc/FTO/C60		1.04	18.91	0.7811	15.42	Ke et al. (2015)
PbPc		0.816	13.4	0.363	3.97	Suzuki et al. (2015)
ZnPc		0.774	10.4	0.435	3.50	
CuSCN		0.604	7.87	0.316	1.50	
CuSCN		1.10	23.25	0.77	20.06	Arora et al. (2017)
Spiro-OMeTAD	Small molecule	1.12	22.5	0.757	19.0	Jeon et al. (2015)
pm-Spiro-OMeTAD		1.01	21.1	0.652	13.9	Jeon et al. (2014)
po-Spiro-OMeTAD		1.02	21.2	0.776	16.7	
pp-Spiro-OMeTAD		1.00	20.7	0.711	14.9	
PST1		1.024	17.63	0.73	13.44	Ganesan et al. (2015)
ATT-OMe		1.07	21.75	0.781	18.13	Zimmermann et al. (2017)
FDT	Polymer	1.148	22.7	0.76	20.2	Saliba et al. (2016)
FA-MeOPh		0.924	18.39	0.698	11.86	Choi et al. (2014)
DMFA-FA		1.002	20.661	0.687	14.21	Choi et al. (2015)
m-MTDATA		1.035	22.5	0.778	18.12	Chen et al. (2018b)
KR131		1.145	20.7	0.77	18.3	Rakstys et al. (2015)
PTAA		1.06	24.7	0.775	20.2	Yang et al. (2015)
TFB		0.96	17.5	0.65	10.92	Zhu et al. (2014)
PFB		0.91	13.8	0.64	8.03	
PFO		0.61	3.6	0.56	1.22	
PPP		1.03	21.6	0.75	16.5	Yan et al. (2015)
PCPDTBT		0.77	10.3	0.667	5.3	Heo et al. (2013)
PCDTBT		0.92	10.5	0.437	4.2	
PTB7-Th/MoO_3_		1.03	14.96	0.75	11.04	Kim et al. (2014)
PBDTTT-C/MoO_3_		0.868	17.68	0.6483	9.95	Chen et al. (2015)
PTB-BO		0.827	14.35	0.62	7.4	Lee et al. (2015)
PDPPDBTE		0.855	14.4	0.749	9.2	Kwon et al. (2014)
P3HT		1.01	20.3	0.80	16.1	Zhang et al. (2018)

**Figure 8:**
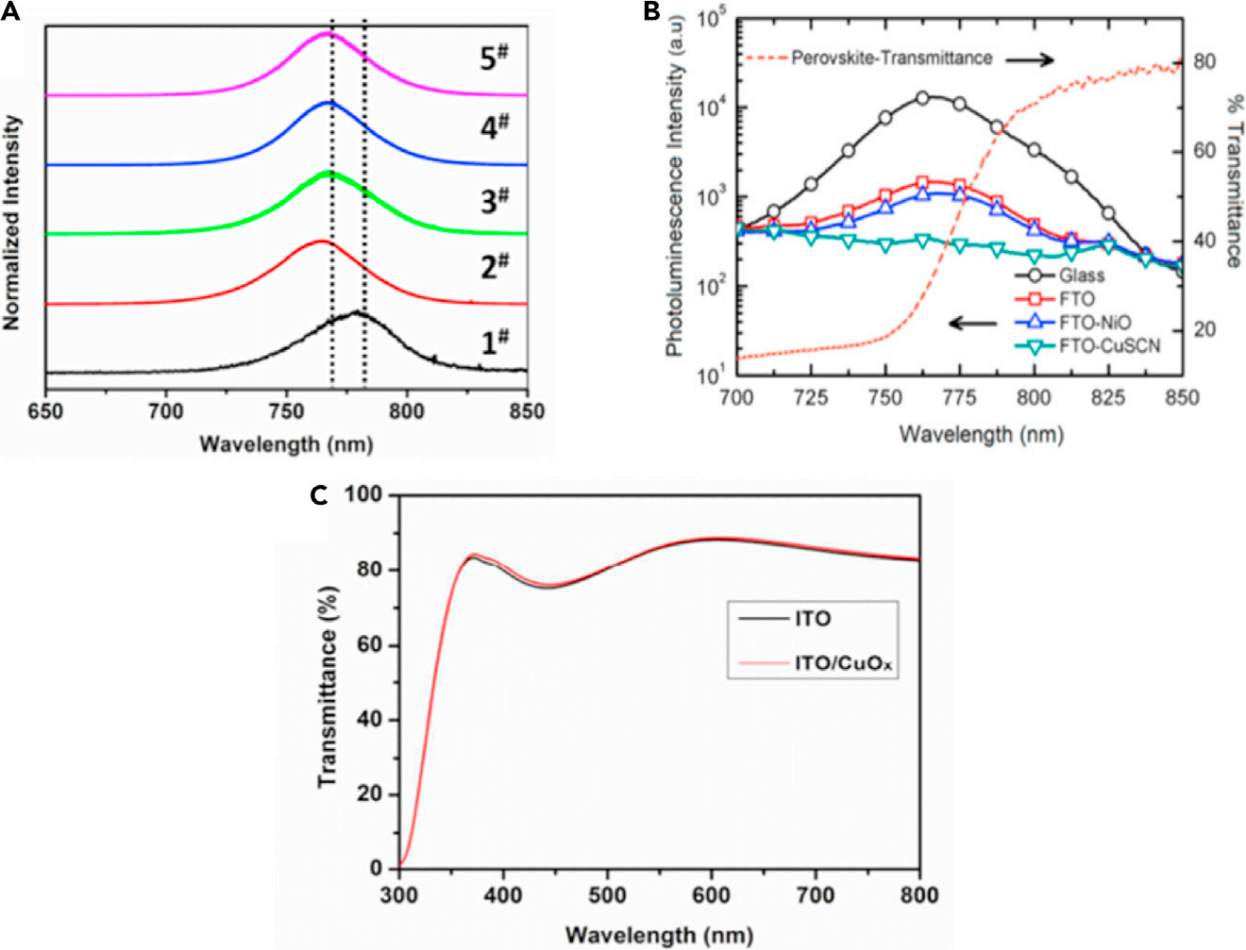
Correlation transmission spectrogram (A) Steady-state photoluminescence (PL) spectrum of NiO_x_ (linear graph): 1#–5# (the concentration of MAI and PbI_2_ is fixed at 1 M, and the ratio of DMSO: PbI_2_ is changed from Adjust from 0:1 to 14.1:1 to keep the total solvent volume at 1 mL) (Bai et al., 2017). (B) Transmission spectrum of CuSCN (Subbiah et al., 2014). (C) Transmission spectrum of CuO_X_(Sun et al., 2016b).

Compared with organic materials, inorganic materials generally have better chemical stability, higher hole mobility, and lower preparation costs, which are of great significance for the preparation of low-cost and high-stability PSC. Among the inorganic hole transport materials, NiO_X_(Bai et al., 2017) is the material with the most stable chemical properties and the best compatibility with halide perovskites. NiO_X_ is a p-type semiconductor metal oxide with a wide bandgap and cubic structure. The material can not only efficiently extract holes from the perovskite material, but its energy level structure matching the perovskite material can also maximize the open-circuit voltage of the cell. Also, there are most Cu compounds such as CuSCN (Subbiah et al., 2014),CuO_X_ (Sun et al., 2016b), etc., as shown in Figure 8 CuSCN has good transparency in the UV, visible and infrared spectrum, a large forbidden bandwidth (Eg = 3.6 eV), high hole mobility (0.01–0.1 cm^2^V^−1^ s^−1^), and relatively fine chemical stability, and can be synthesized by a simple method. Recently, because of its proper band matching with perovskite materials such as MAPbI_3_, CuSCN has shown promise as a high-efficiency material in PSC. Since solvents usually decompose perovskite, inorganic hole materials are mainly used in trans-perovskite cells. Kamat et al. firstly used CuI as a hole material to produce a formal structure cell with an efficiency of only 6% (Christians et al., 2014). The fill factor of the cell is up to 0.70, but the charge recombination is serious, and the open-circuit voltage of the cell is only 790 mV, so the efficiency of the cell is not high. To eliminate the influence of solvents on perovskites, Gharibzadeh et al. used gas-solid reaction to deposit CuI, the efficiency was increased to 7.4%, and the cell short-circuit current was as high as 32 mA·cm^2^ (Gharibzadeh et al. 2016). Sun et al. prepared trans-perovskite cells by spin-coating CUI, its efficiency reaches 16.8% (Sun et al., 2016a). Ito et al. introduced CuSCN (Qin et al., 2014), which has high transmittance in the visible and near-infrared regions. The bandwidth is 3.6 eV and the hole mobility is very high, and the cell efficiency of CuSCN prepared based on the knife coating method is 12.4%. Subsequently, Bian et al. electroplated CuSCN with a thickness of 57 nm in the trans-plate structure perovskite cell and then deposited MAPbI_3_ with a one-step method, which increased the cell efficiency to 16.6% (Ye et al., 2015). Zhu et al. introduced CuInS_2_ quantum dots to prepare a perovskite cell with a conversion efficiency of 8.4% (Li et al., 2015). Bhattacharyya et al. introduced Sb and S, synthesized the ternary oxide CAS (Cu_12_Sb_4_S_13_) as a hole transport material to obtain a flat panel cell with a conversion efficiency of 6.5% (Tamilselvan and Bhattacharyya, 2018), and an effective area of 0.09 cm^2^.NiO_X_ is a material with high transmittance, with a bandwidth of 3.5–3.9 eV. Sarkar et al. electrodeposited NiO_X_ thin films in trans-plate perovskite cells and then used UV light or plasma to clean the surface of the film to improve photovoltaic performance, achieving a cell with a conversion efficiency of 7.26% (Subbiah et al., 2014). Seok et al. used pulsed laser deposition to prepare nano-NiO_X_ thin films (Park et al., 2015) the cell efficiency increases to 17.3%, and the fill factor is as high as 0.813. Jen et al. used Cu doped NiO_X_ to improve the conductivity of the hole layer and obtained a cell with a conversion efficiency of 17.8% (Jung et al., 2016). Yue et al. used Cu doped NiO_X_ and zirconium acetylacetonate to modify the aluminum electrode, and the efficiency of the trans-perovskite cell prepared reached 20.5% (Yue et al., 2017). The photovoltaic parameters of various inorganic hole-transporting PSC are shown in Table 2. Although there are many types of inorganic hole transport materials, the reported performance of devices based on inorganic hole transport materials is lower than that of organic hole transport materials. Organic materials have better conversion efficiency than inorganic materials in terms of solar light absorption. This may be due to the energy bandwidth of the inorganic material itself, or its structure. This needs further exploration and research. By optimizing the composition and structure of inorganic materials, the photovoltaic performance of PSC is improved.

**Table 2:** Comparison of photovoltaic parameters of various inorganic hole-transporting PSC

Inorganic materials	Voc(V)	*J*_sc_ (mA cm^−2^)	FF	PCE(%)	References
CuI	0.55	17.8	0.62	6.0	Christians et al. (2014)
CuI(gas–solid transformation method)	0.73	32.72	0.31	7.4	Gharibzadeh et al. (2016)
CuI(facile solution-processed method)	1.01	22.9	0.728	16.8	Sun et al. (2016a)
CuSCN	1.01	19.7	0.62	12.4	Qin et al. (2014)
CuSCN(electrodeposited)	1.00	21.9	0.758	16.6	Ye et al. (2015)
CuInS_2_	0.92	18.6	0.487	8.4	Lv et al. (2015)
NiOx	0.78	14.2	0.65	7.26	Subbiah et al. (2014)
CAS	0.80	18.08	0.80	6.5	Tamilselvan and Bhattacharyya (2018)
NiOx (PLD method)	1.06	20.2	0.813	17.3	Park et al. (2015)
Cu: NiO_X_	0.88	20.23	0.76	17.8	Jung et al. (2016)
Cu- NiO_X_	1.13	23.49	0.77	20.5	Yue et al. (2017)

**Figure 9:**
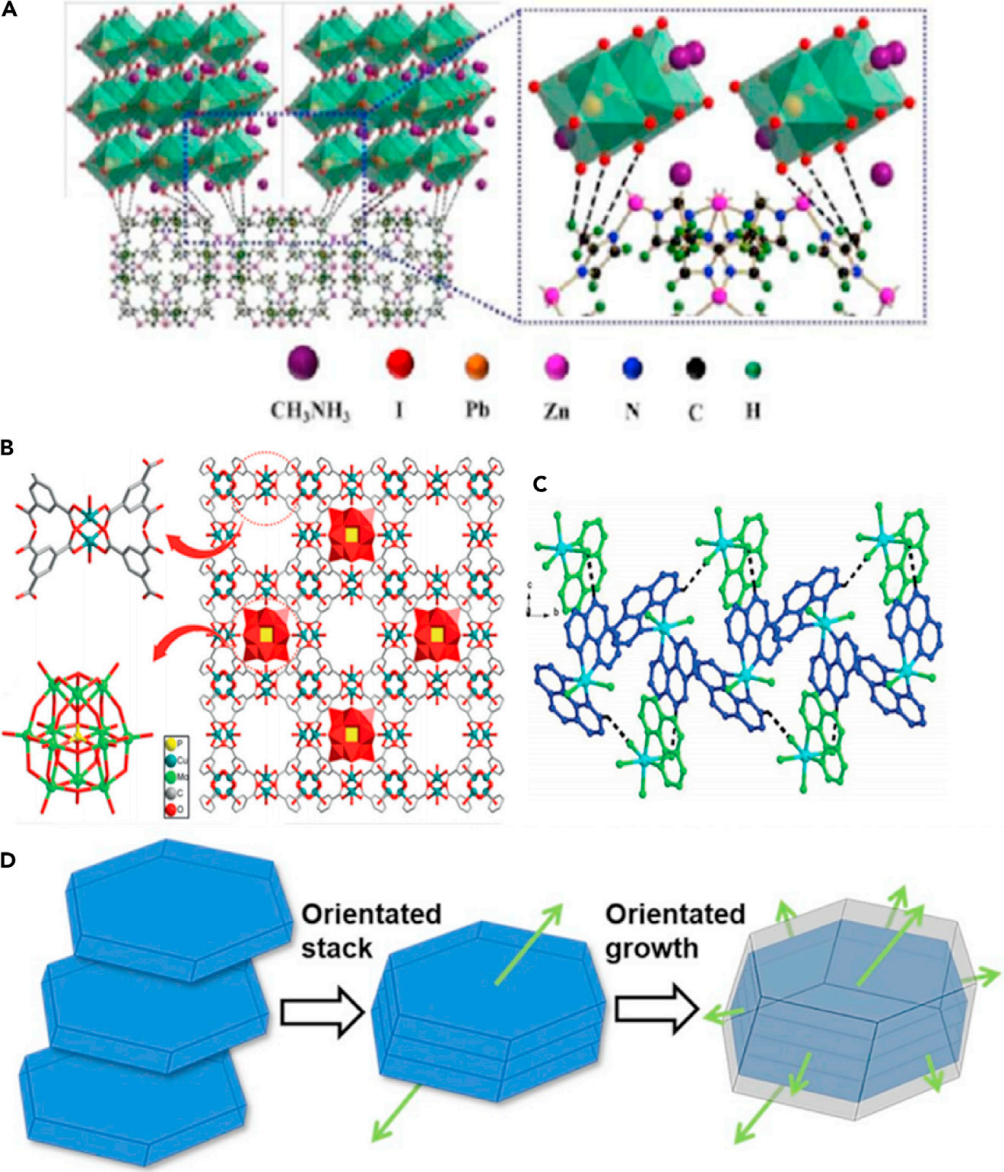
The 3D structure of various MOFs applied in the hole transport layer (A) CuO@NiO HTL (Shen et al., 2018). (B) POM@Cu-BTC HTL (Dong et al., 2019). (C) HTM/In2 (Li et al., 2018). (D)SP-MOF (Huang et al., 2019).

It can be seen from the above that both organic and inorganic hole materials are designing different types of hole structures to improve the photovoltaic performance of PSCs. It is well known that the hole structure of MOF material is unique, so it has been widely developed and applied to the HTL material of PSC. Ghorashi uses MOF as a precursor, using the sacrificial template method (Shen et al., 2018), CuO@NiO nanospheres with core-shell structure were prepared by solvothermal method, and the structure is shown in Figure 9A (Hazeghi et al., 2020). Compared with NiO nanoparticles, core-shell CuO@NiO nanostructures have smaller band gaps and fewer defect states (Chavan et al., 2019; Liu et al., 2019). As a p-type HTL, nanoparticles are used in the manufacture of conventional PSC. Compared with the PSC made of NiO HTL, the photovoltaic characteristics of the PSC with core-shell CuO@NiO HTL exhibit a greater current density (21.80 mA cm^−2^), open-circuit voltage (0.91 V), and photon conventional efficiency (10.11%). The improvement in the efficiency of Cuo@NiO HTL containing PSC may be related to the suppression of non-radiative recombination at the perovskite/HTL interface and fine band alignment between perovskite and HTL, which effectively inject the cavity from the LUMO level of perovskite into the perovskite (Yamada et al., 2016; Basith et al., 2014). Also, the CuO shell on the NiO nanocrystal not only reduces traps and defect states, but also promotes the hole transfer process between the perovskite layer and the HTL, and ultimately promotes the hole transfer process, which leads to an increase in PSC efficiency. Also, Cuo@nio HTL based PSC showed low lag and improved long-term stability, with 39.87% PCE degradation after 1920 hr (68.26% aging after 1248 hr for PSC with SPIRO-OTAD HTL) without encapsulation. Substituting CuO @ NiO HTL for spiro-OMeTAD can realize promising PCE with good long-term stability and lower commercial production price. Similarly, Fan designed and synthesized a mixed oxidant POM@Cu-BTC and applied it to a highly stable PSC. The structure is shown in Figure 9B(Dong et al., 2019). It is found that the hybrid POM@Cu-BTC can not only promote the rapid oxidation of spiro-OMeTAD but also improve the stability of the device. More importantly, the optimally doped devices have enhanced conductivity and carrier extraction at the perovskite/HTM interface, and reduced charge recombination. Benefiting from the doping of hybrid POM@Cu-BTC, the water-resistance of PSC has been improved, and after long-term storage in the environment, PCE retains about 90% of its initial value, which proves that the use of oxidants derived from POM and MOF is a convenient and effective strategy to improve PSC efficiency and environmental stability.

The MOF-In synthesized by Li is used in the HTL of PSC, and the structure is shown in Figure 9C(Li et al., 2018). When In2 is doped in HTL, the corresponding PSC has achieved satisfactory improvement in terms of photoelectric performance. At the same time, the short-circuit current is increased, and the open-circuit voltage and FF are improved. However, due to the limitation of the manufacturing process, the PCE of the controlled PSC is relatively low. Although the device manufacturing conditions are poor, the PCE of the modified PSC has increased significantly from 12.8% to 15.8%. This is mainly due to the positive effect of In2 because it promotes the light absorption of the perovskite and eliminates some undesirable phenomenon (Nguyen et al., 2013). This work proves that MOF can be applied to PSC, which can improve equipment performance and enhance stability. Fan (Li et al., 2019) then incorporated MOF-In10 into the HTL of PSC. Adding In10 can oxidize Spiro-OMeTAD to Spiro-OMeTAD^+^, and the conductivity of the corresponding HTL is greatly enhanced, which facilitates charge transport and inhibits charge recombination in PSC (Farooq et al., 2018). Besides, the visible light of In10 can also help improve the light response of PSC (Xue et al., 2017). Compared with the PSC that does not use In10, the PCE of the modified PSC is enhanced by more than 20%, and the PCE reaches 17.0%, which expands the application range of MOF and opens up a broad road for the development of PSC. Zeng et al. (Huang et al., 2019) combined the metal organic framework material (SP-MOF, as shown in Figure 9D) with the organic Spiro-OMeTAD, and the resulting HTL has a smoother surface, higher hydrophobicity and higher energy level and electrical interface. Due to the directional halo effect, the composite perovskite solar cell exhibits higher conversion power and moisture resistance. The average efficiency of the cell is 13.17%. Even after 9 days in an environment with a relative humidity of 30%, it can still maintain an efficiency of 54% of the initial value.

To solve the PSC stability and power conversion efficiency (PCE) drop caused by perovskite degradation and interface carrier recombination, Zhang et al. (2021) built the dual-functional layer HTM-FJU-17 by incorporating the (Me2NH2)^+^ encapsulated indium-based anionic MOF (FJU-17) as a “capsule” into the HTM. The FJU-17 capsule will passivate organic cation vacancies by releasing (Me2NH2)+ ions, and its anion skeleton can stabilize the positively charged oxidized HTM to improve hole mobility. This proves that the dual-function layer of MOF/HTM can get high-performance PSC. The photovoltaic parameters of the HTL of MOFs applied to PSC are shown in Table 3.

**Table 3:** PV parameters of PSC using MOFs.

MOFs	Device	Voc(V)	Jsc (mA cm^−2^)	FF	PCE(%)	References
MOF-525	Perovskite filma)	0.93 ± 0.02	23.04 ± 1.0	0.60 ± 0.03	12.0 ± 0.5	Chang et al. (2015)
In-BTC	Perovskite filmb)	1.10 ± 0.02	22.99 ± 0.79	0.77 ± 0.03	19.63 ± 1.24	Zhou et al. (2020)
CuO @ NiO HTL(structure)	Hole-transport layer	0.91	21.8	0.51	10.11	Hazeghi et al. (2020)
POM@Cu-BTC HTL	Hole-transport layer	1.11	23.9	0.80	21.44	Dong et al. (2019)
HTM/In2	Hole-transport layer	1.01	21.03	0.74	15.8	Li et al. (2018)
HTM/In10	Hole-transport layer	1.00	24.3	0.70	17	Li et al. (2019)
nTi-MOF	Electron transport layer	1.05	22.61	0.734	18.94(rigid) 17.43(flexible)	Ryu et al. (2018)
MIL-125 (Ti) (structure)	Electron transport layer	1.01	22.81	0.7184	16.56	Hou et al. (2017)
Co-doped Ti-MOF	Electron transport layer	1.027	24.078	0.6495	15.75	Nguyen and Bark (2020a)
m-TiO_2_/ZIF-8	Electron transport layer	0.972	19.8	0.62	12	Chung et al. (2018)
MOF-derived ZnO	Electron transport layer	1.11	22.1	0.74	18.1	Zhang et al. (2019a)
ZIF-8 derived porous carbon skeleton	Electron transport layer	1.06	22.13	0.72	17.32	Zhang et al.(2019b)
mp-TiO_2_/ZIF-8	Interface layer	1.02	22.82	0.73	16.99	Shen et al. (2018)
NiO @ C	Interface layer	1.018	22.394	0.6924	15.78	Nguyen and Bark (2020b)
ZIF-8	Interface layer	1.23	21.8	0.59	16.8	Ahmadian-Yazdi et al. (2020)

a From an average of 40 devices.

b From an average of 20 devices.

### Electron transport layer

The electron transport layer (ETL), as one of the important components of PSC, can be used for the transmission of photo-generated electrons and inhibit the recombination of carriers, which is of great significance for improving the photovoltaic performance of the cell. The role of the ETL is similar to that of the HTL, except that the transported carriers change from holes to electrons. The selection of its materials should follow the following principles: (1) Energy level matching the perovskite absorption layer; (2) Excellent electron mobility, which can reduce the electron transmission resistance and achieve high short-circuit current density;(3) The processability of the solution, used in the manufacture of low-temperature films and compatibility with flexible substrates. In MAPbI_3_, holes have higher mobility than electrons, so compared to the HTL, the ETL is even more indispensable in PSC. Commonly used electron transport materials are also divided into organic and inorganic categories. The most widely used inorganic material is TiO_2_ (Min et al. 2019), which benefits from the perfectly matched energy level structure of TiO_2_ and MAPbI_3_. The most efficient perovskite cell reported in this article is a device based on the TiO_2_ mesoporous structure. Also, SnO_2_(Jiang et al. 2019) and ZnO (Liu and Kelly, 2014) are also commonly used electron transport materials. Inorganic electron transport materials are used in the structure of the forward device because most inorganic materials such as TiO_2_ require high-temperature sintering to obtain a dense transport layer. Also, due to the limitation of solubility, if the spin coating is used, the selectivity to the solvent is less, which is not conducive to the deposition on the perovskite. In reverse structure devices, fullerenes and their derivatives, such as C60 (Dai et al., 2017) and PCBM (Luo et al., 2018), and other organic materials are more common as ETLs. Organic materials have the following advantages: (1) The chemical variability is large, and the molecular structure can be changed through a variety of ways, as to adjust the photoelectric properties of the material and improve the carrier transmission capacity;(2) It is easy to process and can be formed into a large area of the film; (3) The source of raw materials is wide, the price is cheap, and the cost is low; (4) It can be prepared into a flexible film, which can be easily processed into various shapes to adapt to different environments. Fullerene has many unique properties, such as high electron affinity, low recombination energy, and high electron mobility, and can be formed into films at low temperature, can be used for flexible devices, and can inhibit the hysteresis effect of PSC. The LUMO energy level of C60 and PCBM can be matched with the conduction band of the perovskite so that carriers can be effectively separated at the interface of perovskite and fullerene. However, C60 has poor solubility, so it is generally deposited by vacuum evaporation, while its derivatives generally have good solubility and can be prepared by solution spin coating. Besides, small molecule materials such as perylene diimide (PDI) and its derivatives (Castro et al., 2017), naphthalene diimide (NDI), and its derivatives (Yan et al., 2019) have also been widely studied and applied.

**Figure 10:**
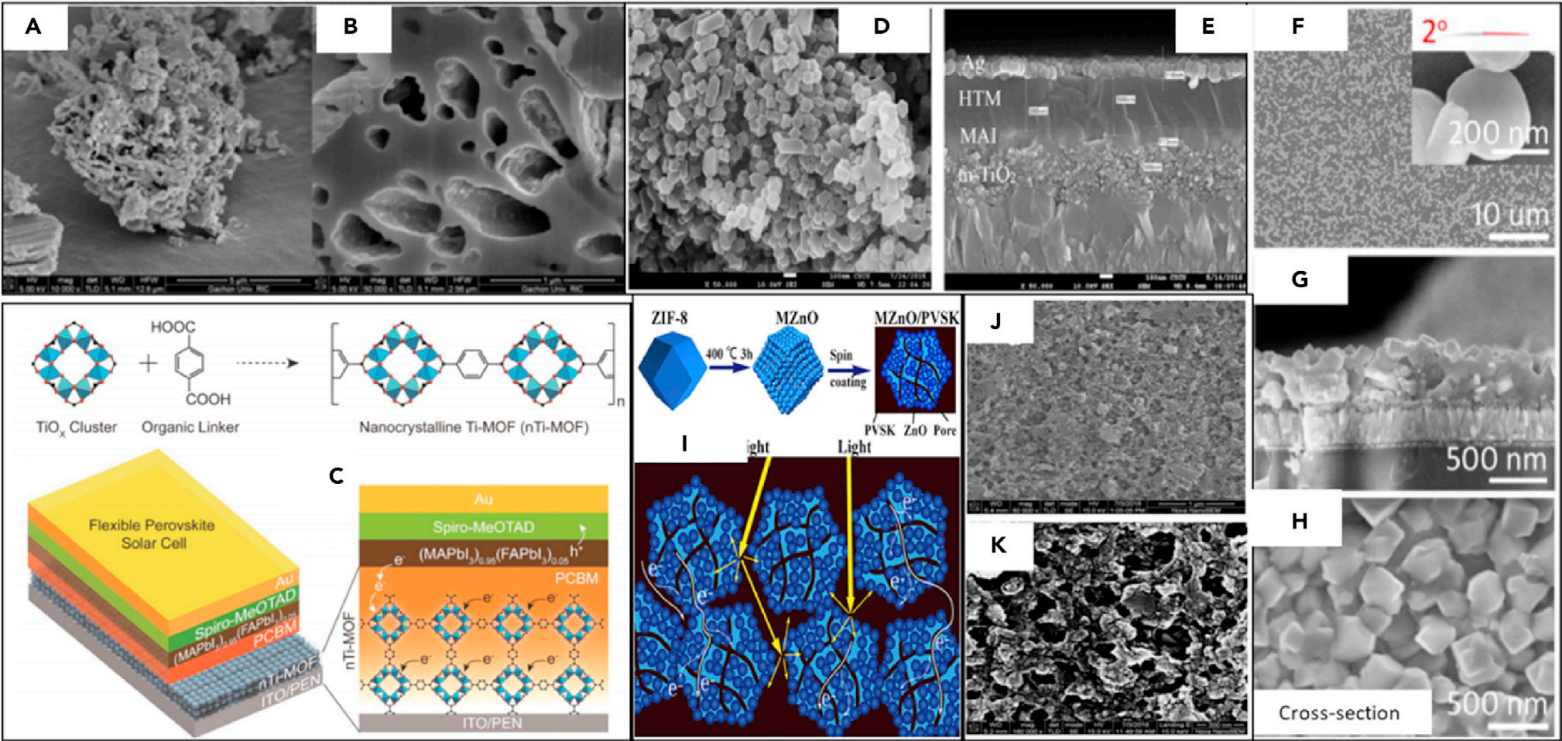
The structure of various MOFs applied to the electron transport layer (A and B) Co-doped Ti-MOF (Nguyen and Bark, 2020a). (C) nTi-MOFs (Ryu et al., 2018). (D and E) m-TiO_2_/ZIF-8 (Chung et al., 2018). (F–H) MIL-125 (Ti) (Hou et al. 2017). (I) MOF-derived ZnO (Zhang et al., 2019a). (J and K) ZIF-8 Derived Porous Carbon Skeleton (Zhang et al., 2019b).

The large band gap allows electrons to be excited and injected, which makes electron transmission inefficient. To solve this problem, it is very important to reduce band gap. Therefore, a method of doping a semiconductor with metal is put forward. Bark successfully synthesized Co-doped TiO_2_ samples by the solvothermal method and used it as an effective ETL to make PSC (Nguyen and Bark, 2020a). Compared with the commercially available dye sol TiO_2_, the solar cell based on the prepared material exhibits enhanced performance. With an open-circuit voltage of 1.027 V, a current density of 24.078 mA/cm^2^, and a fill factor of 64.95%, an excellent PCE of up to 15.75% was obtained. Co reduces the bandgap of TiO_2_ and promotes distortion due to the presence of Co defect atoms in the TiO_2_ lattice (Biswajit and Amarjyoti, 2012). 1 wt% Co-doped TiO_2_-MOF has a higher porosity structure than dye sol TiO_2_, and shows better photovoltaic performance, as shown in Figures 10A and 10B. Due to Co doping, the charge transport resistance (RTRANS) and charge recombination resistance (RREC) is significantly reduced, and the dopant promotes electron transport and eases electron–hole recombination. These results are attributable to the internal and surface morphological rearrangement obtained by the thermal decomposition of the MOF template, and the improvement of electron transport caused by Co doping (Biswajit and Amarjyoti, 2012; Khurana et al., 2015).

Park prepared nanocrystalline nTi-MOF (about 6 nm) and used it to generate effective ETL in PSC (Ryu et al., 2018). nTi-MOFs are highly dispersed in organic solvents to easily produce uniform and ultra-thin films, as shown in Figure 10C. The electronic structure of nTiMOF is suitable for the transport of electrons generated in the perovskite layer of PSC. Doping PCBM into nTi-MOF ETL can improve the conductivity of the film and inhibit the direct contact between the perovskite and the substrate. Therefore, they provide excellent device performance for rigid (PCE = 18.94%) and flexible (PCE = 17.43%) PSC architectures. The results show that nTi-MOF has great potential in designing highly flexible architectures in PSC and will bring excellent device performance.

In addition to the above, the MOF of ZIF-8 can also be used as an intermediate layer between the mesoporous TiO_2_ and the perovskite layer in PSC (Chung et al., 2018). The ZIF-8 solution is dried on the mesoporous TiO_2_ layer to form a very thin ZIF-8 film, which can then act as an additional light-absorbing layer in the short wavelength range of the solar cell, thereby improving the performance of the cell as shown in Figures 10D and 10E as shown. Using the ZIF-8 coated TiO_2_ layer can enhance the incident photon current conversion efficiency (IPCE) of the PSC. When ZIF-8 is present, the conversion efficiency of PSC increases from 9.6% to 12.0%.

In addition to being mixed with interface layer devices, MOFs can also be used in the process of preparing the interface layer to develop novel structural materials. Chen studied a kind of quasi-mesoscopic PSC (QM-PSCs) with porous hierarchical TiO_2_ (hier-TiO_2_) nanostructures as scaffolds (Hou et al., 2017). The porous TiO_2_ nanostructure is synthesized by sintering the MOF MIL-125 (Ti) at 500°C in the air, which partly inherits the ordered porosity of MIL-125 (Ti) as shown in Figures 10F–10H. Ordered titanium dioxide nanostructures are dispersed on the dense TiO_2_ layer, forming a quasi-mesoscopic scaffold that can provide sufficient growth space for perovskite grains and promote the orderly growth of perovskite grains (Grancini et al., 2014). The power conversion efficiency (PCE) of QM-PSC was 16.56%, which was much higher than the PCE (11.38%) of PSC supported by traditional small TiO_2_ nanoparticles (NPT-TiO_2_) and the PCE layer of planar PSC with compact TiO_2_ (6.07%). The PCE of the PSC with hier-TiO_2_ and npt-TiO_2_ remained 47% and 22% of the initial PCE value after 30 days of aging in the air, which indicates that the PSC with the hier-TiO_2_ scaffold showed better stability and moisture resistance. The performance enhancement of QM-PSC is mainly attributed to the quasi-mesoscopic scaffold with good wettability and the ordered porous layer of TiO_2_ nanostructure (Huang et al., 2016). The porous nanostructured materials prepared by the MOF structure help to form high-quality perovskite films with better crystallinity and fewer pinholes, and improve the contact performance between the perovskite and the ETL (Zheng et al., 2015).

Zhang proposed a new strategy to enhance electron transport and extraction in PSC by using MOF-derived ZnO as ETL (Zhang et al., 2019a). The introduction of MOF-ZnO will certainly quench the PL intensity, shorten the electron lifetime, expand the charge recombination resistance and reduce the trap density state. Effective electron extraction and suppressed electron–hole recombination rate, which promotes electron transport and increases *J*_sc_ and FF (Jang et al., 2016; Kang et al., 2016; Yang et al., 2016a). It has been found that MOF-ZnO with a special morphology and a large number of internal pores can induce higher light absorption density and improve the optical utilization efficiency of perovskite, as shown in Figure 10I. The increase of the interface area between MOF-ZnO and PVSK can improve the carrier extraction efficiency (Yang et al., 2016b). This MOF-derived ZnO is used to optimize the interface in PSC and significantly enhance device performance. The generated Voc is 1.11 V, *J*_sc_ is 22.1 mA cm^−2^, FF is 0.74, and PCE is 18.1%. This work can provide diversified options and designs with ideal performance for perovskite-type solar cell ETL.

Zhang prepared ZIF-8-derived porous carbon framework by carbonizing ZIF-8 coated on FTO substrate (Zhang et al., 2019b). The derived porous carbon is buried under a thin layer of TiO_2_ to serve as the ETL of the PSC, as shown in Figures 10J and 10K. The photovoltaic performance of PSC based on porous carbon layers has been significantly improved. The derived carbon layer can be used as a high-speed electron transport channel from the thin TiO_2_ layer to the FTO substrate (Wu et al., 2011). Besides, the specific surface area of the subsequently deposited TiO_2_ thin layer is relatively increased, which increases the contact interface area between the TiO_2_ layer and the perovskite layer. The enhanced performance of PSC is attributed to the increased specific surface area of TiO_2_ thin layers and improved electron transport through ZIF-8-derived porous carbon layers (Xu et al., 2017). In contrast, the conversion efficiency of PSC based on the ZIF-8-derived porous carbon thin layer was 17.32%, which was better than that of PSC without the derived layer. The photovoltaic parameters of the ETL of MOFs applied to PSC are shown in Table 3.

By improving the interface state, the reduced trap state and the smooth surface of the TiO_2_ ETL can improve the PSC performance. Li (Ji et al., 2021) prepared a composite film of polyethyleneimine ethoxylation (PEIE) and a two-dimensional metal–organic framework (2D MOF) based on tellurium benzene to achieve the non-destructive passivation of TiO_2_. The ETL can not only realize the effective passivation of TiO_2_, but also can further improve the morphology of the perovskite film. After modification of the PEIE-2D MOF composite film, the morphology and crystallinity of the perovskite film have been greatly optimized. As the trap states in the TiO_2_ layer are reduced, the electron transport in the device is enhanced, and finally high-efficiency and stable cell performance is realized.

### Interface sandwich

**Figure 11:**
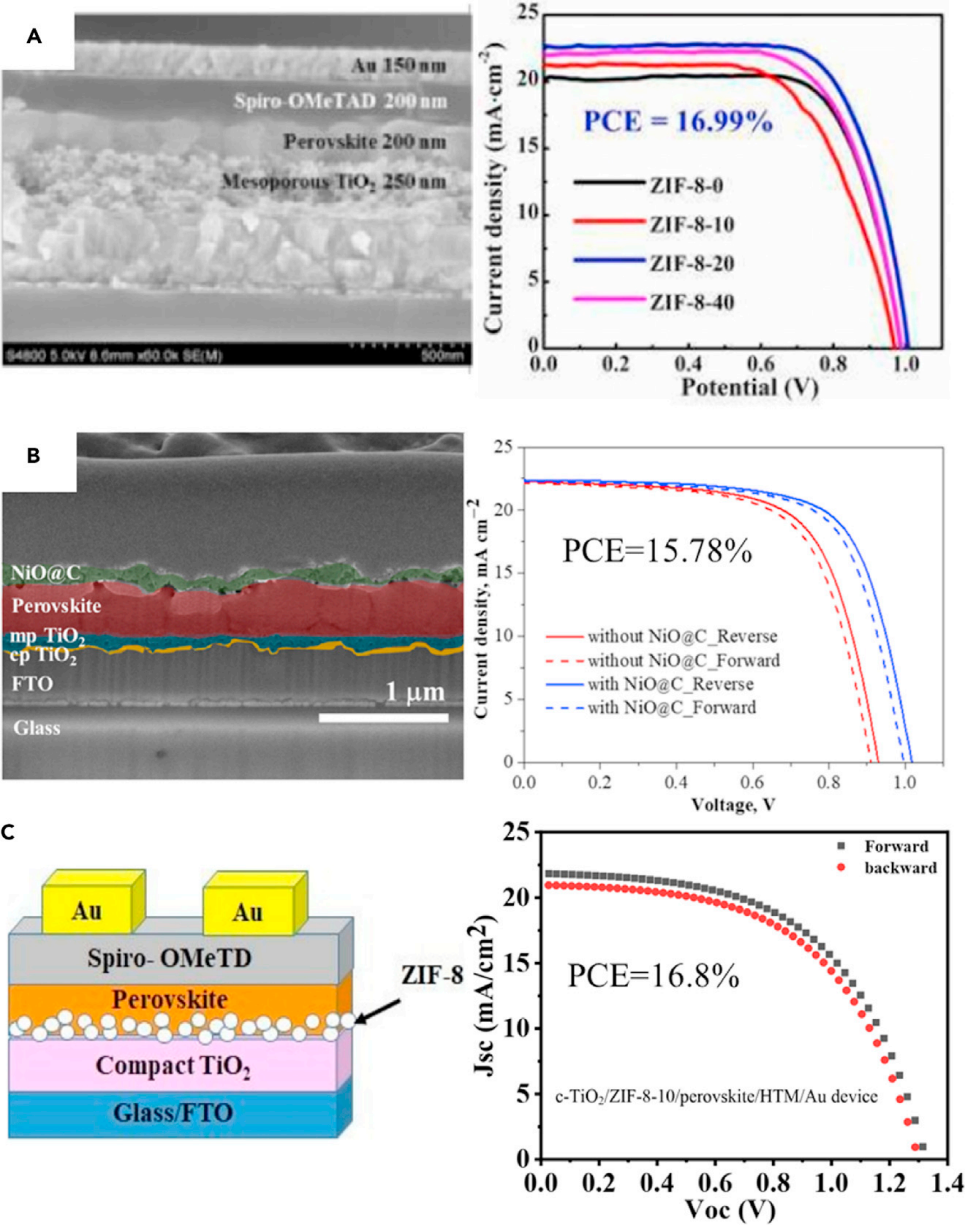
The structure and photovoltaic performance of various MOFs applied to the interface sandwich (A) Mesoporous-TiO_2_/ZIF-8/perovskite/Spiro-OMeTAD/Au device (Shen et al., 2018). (B) NiO@c/perovskite/mp-TiO_2_/cp-TiO_2_/FTO/Glass device (Nguyen and Bark, 2020b). (C) c-TiO_2_/ZIF-8-10/perovskite/HTM/Au device (Ahmadian-Yazdi et al., 2020).

Li (Shen et al., 2018) introduced a metal–organic framework as an interface layer into PSC for the first time, as shown in Figure 11A. Using ZIF-8 to modify the interface effectively enhanced the crystallinity and grain size of the perovskite, and the photovoltaic performance of the PSC was significantly improved, resulting in a maximum PCE of 16.99%. By coating, the ZIF-8 layer on the interface between mp-TiO_2_ and the perovskite film, the crystallinity, and morphology of the perovskite film has been significantly enhanced. Under the optimum conditions, the PCE of MP-TiO_2_ cells coated with ZIF-8 reaches 16.99% at 20 min, which is higher than that of the reference cells without ZIF-8 coating. This is because the extra scaffold can support the perovskite crystallites in orderly arrangement during the initial stage of crystallization (Chang et al., 2015). At the same time, the methyl group in the crystal structure of ZIF-8 can also form hydrogen bonds with the halide anion of the perovskite, which enhances the cohesion of the perovskite film with the substrate (Li et al., 2015). Therefore, an appropriate amount of ZIF-8 can effectively crosslink adjacent perovskite grains, strengthen the perovskite grain size and smooth morphology, and finally form a high-quality capping light-trapping layer on the surface of the mp-TiO_2_ film. These results indicate that the interface between mp-TiO_2_/ZIF-8 and the perovskite film plays an important role, which can effectively inhibit the recombination of photogenerated carriers and improve the extraction of electric charges.

BARK successfully synthesized high porosity MOF derived NiO@c nanoparticles with a BET surface area of 69.89m^2^g^−1^ by solvothermal method. It was subsequently used as an interface layer in planar n-i-p PSC (Nguyen and Bark, 2020b). By inserting NiO@C film between the perovskite layer of PSC and the spiro-OMeTAD layer, the number of defects on the surface of the perovskite reduces the charge transfer efficiency, thereby increasing the power conversion efficiency of the PSC, and the PSC's PCE increased from 13.79% to 15.78%, as shown in Figure 11B.

Eslamian modified the interface layer between c-TiO_2_ and Cs/MA/FA perovskite layer by applying the ZIF-8 layer, thereby improving the crystallinity and photovoltaic characteristics of PSC (Ahmadian-Yazdi et al., 2020), as shown in Figures 11C. By using ZIF-8, the grain size and coverage of perovskite have been significantly improved. The PCE of the best cell is 16.8%, and the average perovskite grain size reaches 522 nm, which is 2.5 times larger than the perovskite grain size obtained on c-TiO_2_ (without ZIF-8). Besides, cells exhibit more effective charge extraction than ordinary cells, which is related to reducing interface defects and improving interface characteristics. This indicates that the interface between c-TiO_2_ and perovskite, which plays an important role in the PCE of PSC, can be effectively modified by the ZIF-8 of the mesoporous MOF to reduce the recombination of photogenerated carriers, thereby increasing the PCE of PSC. Table 3 shows the interlayer photovoltaic parameters of MOFs applied to PSCs.

At present, MOFs are not widely used in PSCs. The main reason is that they are limited by the previous conductivity problems. Therefore, it can only be used as doping in PSCs. Few MOFs are used as a non-doped single-layer structure in cells. At present, only titanium-based and zinc-based MOFs are used as electron transport The layer is applied in the cell.

## Conclusion and outlook

### Conclusions

With the rapid development of PSC, scientists have gradually realized that not only the perovskite layer is an important factor affecting device performance, but also the ETL and HTL cannot be ignored, among which the ETL is crucial. For an effective PSC, the ETL should have great energy level matching to achieve effective charge transfer and hole blocking, high electron mobility, ensuring fast electron transport, high stability and low cost. For PSC with an n-i-p structure, the electron transport materials are mainly organic materials, such as graphene, fullerene, and their derivatives. The advantage of organic ETLs is that they can be easily processed in solution, but their poor environmental stability and poor light stability limit their commercial development.

**Figure 12:**
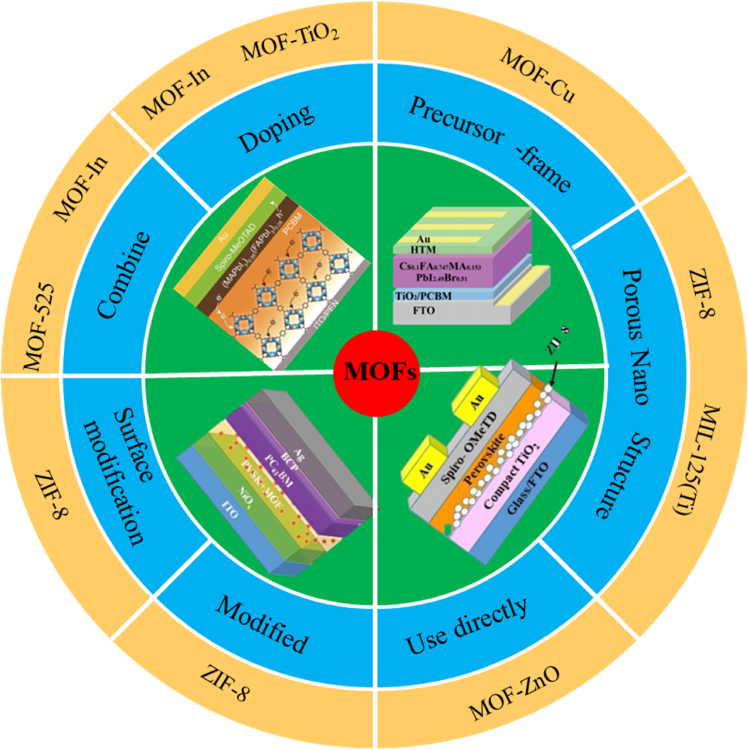
Application distribution of MOFs in PSC.

In this review, the impressive work using MOFs-based materials as functional components of PSC is summarized in detail. These major achievements show that in addition to metal electrodes, MOFs and their derivatives can be applied to various functional layers of PSC. According to the structure of the PSC, we summarized the current applications of MOFs from the perovskite film, HTL, ETL, and interlayer respectively. As shown in Figure 12, due to its high stability and unique semiconductor characteristics, all the above-mentioned MOF-based materials can be developed in perovskite films to enhance the perovskite crystallinity and effectively passivate the perovskite films. At the same time, it is beneficial to charge transport and inhibits charge recombination in the PSC, so that the conductivity of the HTL is greatly enhanced. In terms of structure, nanoparticles modified by MOFs and MOFs composites still maintain the original framework, and MOF framework with high specific surface area, porous structure helps to form a high quality ETL with better crystallinity and fewer pinholes, to strengthen the electronic transmission of the PSC, and to improve the contact properties between perovskite and ETL. However, to improve the conversion efficiency and stability of MOFs-based materials for PSC, some new strategies and challenges still need to be further considered. We have raised these questions below, and we believe that future developments on these topics will broaden our understanding of MOF and PSC.

### Outlook

For PSC with an n-i-p structure, the metal oxide is the most commonly used choice for the ETL. TiO_2_ was the first to be used. In 2009, PSC using mesoporous TiO_2_ as ETL was reported for the first time. So far, TiO_2_ is still the first choice as the ETL. However, scientists have gradually realized some shortcomings of TiO_2_ (Shariatinia, 2020; Kim et al., 2021). For example, the low electron mobility of TiO_2_ and its surface adsorption of oxygen and UV are likely to affect the long-term stability of perovskite cells and limit the further improvement of device efficiency. As a result, many other semiconductors have been studied as potential candidates to replace TiO_2_, such as SnO_2_ (Guo et al., 2020a), ZnO (Bhatt et al., 2021), CdSe (Qi et al., 2018), Zn_2_SnO_4_ (Sadegh et al., 2020), etc. While these materials have advantages, they also show some disadvantages. For example, ZnO has a high electron mobility of 205–200 cm^2^V^−1^s^−1^ and high conductivity due to the self-compensation effect, but ZnO easily reacts with perovskite, which results in the poor long-term stability of the device. However, the preparation process of Zn_2_SnO_4_ is cumbersome. CdSe is easy to prepare using a low-temperature solution method, but the high toxicity of cadmium is worrying, which makes it necessary and critical to treat liquid waste containing cadmium solutions. Although significant progress has been made in various ETLs to replace TiO_2_, as mentioned above, there are still problems such as low device efficiency, poor chemical stability, or high-temperature processing (Shariatinia, 2020; Zy et al., 2020; Salhi et al., 2018). For the development of MOFs materials, it has been developed from a single porous structure and large specific surface area to the semiconductor characteristics of n-type and p-type conductive MOFs. As the materials themselves are prepared by various methods, MOFs can be prepared simply to make up for the vacancy of the HTL in PSC. Then, it is of great significance to find out how to prepare a kind of p-type MOFs with high film-forming quality and high mobility at low temperature.

Perovskite films will undergo large-scale ion migration under external light or electric fields, which is one of the unavoidable problems of the perovskite material itself. Ion migration will produce a large number of vacancies and substitution defects in the perovskite film, which will reduce the photoelectric properties and stability of the film. At the same time, it will also change the potential distribution inside the cell, affect the carrier transmission efficiency, and greatly affect the perovskite film and the stability of the device. Therefore, according to the factors of perovskite ion migration, the control of ion migration is realized through modification and modification design of perovskite materials, which is of great significance to improve the performance and stability of perovskite devices. However, the role of MOFs cannot be fully reflected in the doped form, so we are looking for an MOF material that can directly use MOFs as a non-doped pure phase substance and as a HTL. At present, we analyze the vacancies in this field mainly because of the following two aspects: On the one hand, the MOFs themselves are almost all insulating, and it is difficult to greatly enhance the conductivity through structural control and functional group modification. On the other hand, although some MOFs materials are p-type materials, they are either limited by the material’s inability to form films or poor film quality, so they can only be used in devices in the form of doping, or, although the film can be formed the energy level structure matching is too poor to extract holes from the perovskite layer and transport the holes to the electrode. For the development of MOFs in PSC, future research can be conducted according to the following directions:

(a) Utilization of MOFs performance. On the one hand, MOFs have great untapped potential in magnetism, fluorescence, nonlinear optics and other physical and chemical directions. With the development of new materials, try to utilize more of the various MOFs that can improve the photovoltaic performance of PSC. At present, the use of MOFs mainly focuses on its modification and optimization of each component of PSC, which shows its excellent effect on a single component. However, the application of the whole PSC is still lack of research.
(b) Utilization of MOFs structure. MOFs have abundant functions due to their porous nanostructures, and each component of PSC can make use of the unique characteristics of this structure to conduct innovative research, which will be an interesting attempt. Due to the wide variety of materials and derivatives of MOFs, it is impossible to develop and test each sample, but as the heat gained in this cross direction increases, more gaps will be filled.
(c) Development of new materials. Due to the wide variety of materials and derivatives of MOFs and perovskites, it is impossible to develop and test each sample, but as the heat gained in this cross direction increases, more gaps will be filled.

Although the application of MOFs-based materials to PSC is still in its infancy and there are few related articles, the known researches prove that they can play an effective role in the functional components of PSC. With the current rapid development of MOFs and PSCs, we believe that the application of MOF-based materials to PSC will receive more attention and accomplishments, which will greatly accelerate the development of this intersecting field.
